# Diagnosis, treatment and clinical outcomes of extrauterine sites of leiomyomatosis: a systematic review

**DOI:** 10.1080/07853890.2025.2546681

**Published:** 2025-08-21

**Authors:** Jacopo Di Giuseppe, Leonardo Natalini, Mila Bordini, Daniele Crescenzi, Linda Grementieri, Jessica Petrucci, Arianna Asaro, Luca Giannella, Giovanni Delli Carpini, Camilla Grelloni, Andrea Ciavattini

**Affiliations:** Obstetrics and Gynecologic Section, Department of Odontostomatologic and Specialized Clinical Sciences, Università Politecnica delle Marche, Ancona, Italy

**Keywords:** Hysterectomy, Leiomyoma, laparoscopy, laparotomy, myomectomy, morcellation

## Abstract

**Background:**

This review aims to provide a comprehensive description of the clinical presentation, treatment, and histopathological features of extrauterine sites of leiomyomatosis (ESL), such as disseminated peritoneal leiomyomatosis (DPL), parasitic myoma (PM), benign metastatic leiomyoma (BML), and intravascular leiomyoma (IVL). The impact of previous surgery (hysterectomy or myomectomy) on development of intra-abdominal extrauterine leiomyomas (IAELs) and extra-abdominal extrauterine leiomyomas (EAELs) has been evaluated.

**Methods:**

According to PRISMA guidelines, we searched PubMed, Scopus, and Web of Science databases. Relevant articles were retrieved in full-text format and screened based on predefined inclusion and exclusion criteria.

**Results:**

358 studies (372 cases) are included. Among IAELs, the most common symptom is abdominopelvic pain (63.9% of DPL, and 69.2% of PMs cases, respectively). In contrast, EAELs exhibit heterogeneous clinical presentations; dyspnea is the most common symptom (29.7% of BML, and 29.9% of IVL cases, respectively). 68.8% of DPL, 60% of PM, 89.7% of BML, and 56.4% of IVL patients underwent previous uterine surgery. A significant association (*p* = 0.0417) between the type of previous uterine surgery (hysterectomy and myomectomy) and the subsequent location of ESLs is found (68.7% VS 51.4% in EAELs, 31.3% VS 48.6% in IAELs, respectively).

**Conclusions:**

DPL, PM, BML, and IVL exhibit overlapping characteristics, requiring a multimodal approach that includes imaging, histopathology, and surgical or medical management. Greater awareness among clinicians is needed regarding surgical procedures involving ligation and/or cutting of major uterine vessels, which appear to contribute to the development of EAELs, and morcellation, which tends to promote intraperitoneal metastatic spread.

## KEY MESSAGES

- Disseminated peritoneal leiomyomatosis, parasitic myoma, benign metastatic leiomyoma, and intravascular leiomyoma represent a continuum of extrauterine sites of leiomyomatosis with overlapping but distinct features, requiring careful diagnostic differentiation.
- While surgical excision remains the mainstay, an integrated approach including imaging, histopathology, and medical-surgical management is essential for optimal patient outcomes.

## 1. Introduction

Uterine leiomyomas represent the most prevalent benign neoplasms within the female genital tract [1,2]. However, in certain instances, extrauterine fibromatous lesions may develop, such as benign metastatic leiomyoma (BML), parasitic myoma (PM), disseminated peritoneal leiomyoma (DPL), and intravascular leiomyoma (IVL). Although they apparently maintain the same histopathological characteristics of benign uterine leiomyomas, the etiology of these extrauterine conditions remains uncertain, and several theories have been postulated to explain their origin.

One potential explanation is hormonal-based growth, similar to that of uterine leiomyomas [3–7]. However, the role of iatrogenic spread is increasingly considered central. The theory of hematogenous spread is supported by cases of distant lesions, such as BMLs, and IVLs, in which smooth muscle cells could migrate through the bloodstream and implant in extrauterine organs [3,8]. On the other hand, the theory of direct postsurgical spread is particularly relevant for intra-abdominal extrauterine leiomyomas (IAELs). In this context, surgical morcellation has been associated with the accidental spread of smooth muscle fragments, which may subsequently proliferate to form new peritoneal nodules or PMs [9–12].

The rarity of these pathologies has resulted in a paucity of literature on the subject, which hinders our ability to comprehensively understand their origin and associated risk factors. Consequently, the present study aims to contribute to the existing body of knowledge by conducting a systematic review that analyses the anamnestic, surgical, and histopathological data of affected patients, paying particular attention to the impact of previous surgery and the differences between IAELs, and extra-abdominal extrauterine lesions (EAELs).

## 2. Types of extrauterine sites of leiomyomatosis (ESL)

### 2.1. Disseminated peritoneal leiomyomatosis (DPL)

DPL is a rare benign disorder characterized by the development of multiple round nodules of smooth muscle tissue on the peritoneal surface, and within the subperitoneal space. These nodules can affect any organ in the abdominal cavity, including the retroperitoneum, and range in size from a few millimeters to several centimeters [7,13].

#### 2.1.1. Epidemiology

DPL is extremely rare; it was first described by Wilson and Peale in 1952 [14]. The condition primarily occurs during reproductive age, although cases have also been reported in postmenopausal individuals [7]. Approximately, 100 cases of DPL have been documented in the literature, but the true prevalence remains unclear, as many patients are asymptomatic, leading to frequent underdiagnosis [7].

#### 2.1.2. Pathogenesis

The pathophysiology of DPL remains incompletely understood, though several theories have been proposed. One hypothesis suggests that the nodules of smooth muscle tissue may arise from metaplasia of multipotent mesenchymal cells in the submesothelial layer [15]. These cells, which express estrogen receptors (ERs) and progesterone receptors (PRs), are stimulated to proliferate by both endogenous and exogenous hormones [7]. Additionally, DPL nodules may originate from smooth muscle cells of uterine leiomyomata, which could spread *via* blood vessels or through iatrogenic dissemination during surgical procedures. The established link between DPL and prior gynecological surgeries, particularly laparoscopic hysterectomy or myomectomy involving tissue morcellation, further supports this theory. Indeed, a history of laparoscopic hysterectomy or myomectomy with morcellation is considered a significant risk factor, with the incidence of DPL following these procedures estimated at 0.12–0.9%. Consequently, some researchers have coined the term ‘Post-morcellation parasitic leiomyomatosis’ [12]. Furthermore, a case of familial clustering of DPL has been recently reported, suggesting a possible autosomal-dominant mode of inheritance [16].

#### 2.1.3. Diagnosis

In most cases, DPL is asymptomatic. When symptoms occur, they tend to be vague and nonspecific, including abdominal distension, pain, discomfort, or organ compression due to growing masses, which may even lead to bowel obstruction [7]. Although DPL is sometimes diagnosed following the appearance of these symptoms, it is not uncommon for the condition to be discovered incidentally during preoperative imaging or abdominal surgery for other reasons [17].

When DPL is suspected, imaging studies such as abdominal ultrasound (US), computed tomography (CT), and magnetic resonance imaging (MRI) may be helpful, but a definitive diagnosis requires biopsy and histological analysis. The primary differential diagnosis is late-stage cancer with peritoneal metastasis or primary peritoneal cancer, which can be difficult to distinguish from DPL based solely on imaging [18].

#### 2.1.4. Variants

DPL is classified into two variants, based on different pathogenetic mechanisms. Classical (or primary) DPL occurs in patients without prior uterine surgery, typically due to an estrogenic stimulus, such as oral contraceptives, pregnancy, or an estrogen-secreting tumor. On the other hand, post-morcellation DPL develops in individuals with a history of myomectomy or hysterectomy with morcellation, where estrogenic stimuli are often absent [12,13]. Peritoneal nodules in post-morcellation DPL are typically larger but fewer in number, and less hormone-dependent compared to those in classic DPL. Furthermore, histologically, post-morcellation nodules often resemble the original leiomyomas [13].

#### 2.1.5. Treatment

Due to the rarity of DPL, there are no established treatment guidelines, and management must be tailored to the severity of symptoms, the extent of the disease, and the patient’s age, and performance status [7]. Spontaneous regression of DPL has been reported following cessation or reduction of estrogenic stimuli, such as postpartum, oophorectomy, or discontinuation of hormone therapy [18]. In the absence of malignancy, a conservative approach is often recommended. Medical treatments aimed at reducing estrogen excess include gonadotropin-releasing hormone (GnRH) agonists, aromatase inhibitors (AI), selective estrogen receptor modulators (SERMs), and selective progesterone receptor modulators (SPRM) [19]. Surgical intervention is typically reserved for patients with symptomatic DPL who do not respond to medical therapy or for those at high risk of malignant transformation. The surgical approach usually involves total abdominal hysterectomy (TAH) (if the uterus was preserved), bilateral salpingo-oophorectomy (BSO) to reduce estrogenic stimulation, and debulking of the nodules [17]. A possible correlation between unconfined morcellation of uterine or myomatous tissue and the onset of DPL has been described [20–22]. However, it is important to note that avoiding morcellation may limit the benefits of laparoscopic surgery, such as reduced invasiveness, fewer complications, faster recovery, and earlier discharge [12]. A reasonable compromise is the use of devices designed for protected morcellation, such as laparoscopic bags. In cases where unconfined morcellation is necessary, low-velocity devices should be used, and thorough abdominal cleaning should be performed to remove any tissue remnants [23].

#### 2.1.6. Prognosis and follow-up

In line with the pathogenetic classification, classic DPL typically responds well to medical therapy aimed at estrogen deprivation, while post-morcellation DPL more often necessitates surgical intervention [13]. Although DPL is a benign condition with a generally favorable prognosis, malignant degeneration is rare but it has been reported, with an estimated risk of 2–5% [24,25]. The risk of recurrence remains unclear due to limited data [7].

Patients with DPL should be monitored with US or CT scans to assess for regression, progression, or recurrence, particularly if medical or conservative treatment is chosen. Although the exact duration is not well defined, follow-up should be extended, given the lack of clear guidelines [18]. In cases of recurrence after surgery, a repeat surgical resection of nodules may be considered, though medical therapy with GnRH agonists is often the preferred approach [19]. Diagnosis, treatment, and follow-up of DPL cases are summarized in Box 1.

Box 1. Diagnosis, treatment, and follow-up of disseminated peritoneal leiomyomatosis (DPL).

**Table:** 

**Disseminated peritoneal leiomyomatosis**				
**Clinical presentations**	**Diagnostic tools**	**Differential diagnosis**	**Treatment modalities**	**Follow-up**
**Symptoms** Abdominal/ pelvic pain (most common) Compression of adjacent organs **Signs** Pelvic mass Abdominal distension Vaginal bleeding **Asymptomatic** > 1/3 of cases	**Abdominal US and/or TVUS** Most common initial imaging modality used, low accuracy in distinguishing benign and malignant lesions **Abdominal CT** To assess the extent, sites, and sizes of lesions **Abdominal MRI** To better characterize the soft tissues of lesions **PET-CT/MRI** If malignancy is suspected **Radiologic guided-biopsy** It should be avoided due to low diagnostic accuracy **Histopathology** To obtain definitive diagnosis	Peritoneal carcinomatosis Endometriosis with deep infiltration or abdominal wall involved GISTs Peritoneal tuberculosis Sarcomatosis	**Multidisciplinary discussion** To determine the best therapeutical approach, the potential surgical radicality, and patient’s operability **Debulking surgery** Most effective *LPT vs. LPS* Depending on position, numbers, and size of masses *Morcellation* It should be avoided, containment bags should be used **Hormonal treatment** Primary DPL usually has better response; it should be considered in case of patients unfit for surgery or as maintenance therapy after surgery	**Not-well defined** *US and/or CT* To evaluate regression, progression, or recurrence **Re-excision** It may be considered in case of residual lesions, or recurrences
US: ultrasound; TVUS: trans-vaginal ultrasound; CT: computed tomography; MRI: magnetic resonance imaging; PET: positron emission tomography; GISTs: gastrointestinal stromal tumors; LPT: laparotomy; LPS: laparoscopy.				

### 2.2. Parasitic myoma (PM)

PMs are defined as rare extrauterine smooth muscle neoplasms. International Federation of Gynecology and Obstetrics (FIGO) classificates PMs as ‘type 8’ leiomyomas with no myometrial involvement nor uterine attachment, and which receive blood supply from other abdominopelvic structures to which they have adhered [6,26–28]. PMs can be either primary (spontaneous) or secondary (iatrogenic) [6,28]. Although both DPL and PM are located on peritoneal or subperitoneal surfaces, PMs usually present as solitary, well-demarcated, and larger lesions, in contrast to DPL, which manifests as numerous, small nodules disseminated across the entire abdominal cavity [7,29].

#### 2.2.1. Pathogenesis

Primary PMs are extremely rare and can originate from a pedunculated subserosal leiomyoma that loses its uterine blood supply and survives by obtaining vascularization from other abdominal organs. Another pathogenetic theory is the peritoneal metaplasia, which describes the development of myomas in unexpected areas of the abdomen [6,10,30].

In the last few decades, the iatrogenic etiopathogenetic theory has emerged. Secondary PMs usually originate after laparoscopic myomectomy or hysterectomy that require morcellation of the surgical piece to be extracted through a small incision. This leads to the possible dispersion of myomas fragments in the peritoneal cavity that can regrow as PMs [24,27,28].

It can be assumed that laparoscopic morcellation plays a substantial role in PM pathogenesis since the incidence of this gynecological disorder has increased in the last years in association with the emergence of minimally invasive surgical techniques. In detail, the incidence of iatrogenic PMs after laparoscopic surgery with the use of morcellation and after laparoscopic myomectomy is estimated to be 0.12–0.95%, and 0.20–1.25%, respectively. The diagnosis of this disorder is usually made between three and eight years after the primary surgery [29,30].

#### 2.2.2. Diagnosis

The clinical signs and symptoms are usually nonspecific and correlate with the location and size of the masses. Commonly, PMs are diagnosed incidentally during examinations or surgeries performed for other reasons and are completely asymptomatic. On the other hand, they can present as palpable masses or appear with compression symptoms of the adjacent organs like frequent urination, constipation, abdominal distension, hydronephrosis, abdominal and pelvic pain, and dyspareunia.

Most frequently, the disease occurs in areas that guarantee a good blood supply, which include the entry points of laparoscopic surgery, the broad ligament of the uterus, the abdominal peritoneum, the pouch of Douglas, the sigmoid colon, and the greater omentum [10,29].

The diagnosis of PMs is challenging because IAELs can be confused with malignancy due to their atypical locations, growth patterns, and sizes. When a pelvic mass is found, physical examination and US are the first diagnostic tools. The latter is widely accessible modality for evaluating the morphological characteristics of these pelvic lesions [31]. At US, PMs are usually more hypoechogenic than usual myomas. Additionally, leiomyomas usually show degeneration or central necrosis on CT scans only when they grow sufficiently large, while PMs appear heterogeneous and with hypoattenuation areas even at smaller sizes. This characteristic may result because of the hypoxia experienced by the tissue before obtaining the blood supply from the peripheral and adjacent organs [27,32].

In addition, MRI may be helpful to distinguish between leiomyomas and other solid tumors in the pelvis. Occasionally, PMs may be difficult to differentiate from leiomyosarcomas (LMSs). Therefore, clinical history is particularly important [33,34].

This disorder should be considered in any individual with a pelvic tumor with a history of laparoscopic myomectomy or hysterectomy using morcellation. However, it is also important not to forget that many cases of PMs are not associated with previous uterine surgery [10,29]. A final diagnosis can only be made with histological examination [32].

Differential diagnoses other than LMS are ovarian tumors, tubo-ovarian mass, broad ligament cysts, DPL, when multiple PMs are present, IVL, BML, desmoid tumors, dracunculiasis of the broad ligament or fibrosarcoma [11,27].

Further, PM may be confused with a mass of intestinal origin when attached to the rectosigmoid mesentery. In these circumstances, the measurement of tumor markers, such as CA 125, CA 19-9, or alpha-fetoprotein, will not be of much help since PMs are sometimes associated with elevated levels of these markers and with ascites [29,35].

#### 2.2.3. Treatment

Some techniques can be performed during the primary surgery to avoid or limit fragmentation and dissemination during specimen removal. One option to minimize the risk of iatrogenic PMs could be the morcellation in a containment bag [36]. Furthermore, prior to completing the surgery, myoma remnants in the abdominal cavity should be carefully removed by performing abundant irrigation with normal saline associated with a concomitant reverse Trendelenburg position [9,35,37]. In most cases, PMs can be removed by laparoscopy [29]. An abdominal approach is indicated if the location or size make a laparoscopic approach unsuitable or if malignancy is suspected. When a PM is found in neighboring organs, a multidisciplinary team should be involved to ensure the effective removal of all diseased tissue. Also, the use of robotic surgery has been described with good results [5,28,34].

When the surgical excision is risky due to the macroscopic characteristics of the lesion, medical management can be attempted. There is no consensus on the preferred method for treating and preventing the recurrence of PMs, but AI and SPRM seem to have a significant effect on the reduction of myoma-implantations in comparison to GnRH agonists and SERM [10,34].

Complications are infrequent. Among these, it can be mentioned that the torsion of PM causes acute abdominal pain. An early diagnosis is important because ischemia or necrosis can cause consumptive coagulopathy, gangrene, and peritonitis, which can be life-threatening [4,33]. Other complications can arise as a consequence of the mass effect. In literature are described cases of small bowel obstruction and PMs presenting as an inguinal hernia [27].

Box 2 provides a summary of diagnosis, management, and follow-up of PM cases.

Box 2. Diagnosis, treatment, and follow-up of parasitic myoma (PM).

**Table:** 

**Parasitic myoma**				
**Clinical presentations**	**Diagnostic tools**	**Differential diagnosis**	**Treatment modalities**	**Follow-up**
**Symptoms** Abdominal/ pelvic pain (most common) Compression of adjacent organs Dysmenorrhea **Signs** Pelvic mass Abdominal distension Vaginal bleeding **Asymptomatic** <10% of cases	**Abdominal US and/or TVUS** Initial evaluation but limited specificity **Pelvic MRI** Preferred for better characterize extent, and consistency **Abdominal CT** To assess the extent, to exclude the presence of other abdominal lesions **PET-CT/MRI** If malignancy is suspected **Histopathology** To obtain definitive diagnosis	Ovarian lesions Leiomyosarcoma Peritoneal carcinomatosis Endometriosis with deep infiltration GISTs Peritoneal tuberculosis Sarcomatosis	**Multidisciplinary discussion** To determine the best therapeutical approach, the potential surgical radicality, and patient’s operability **Debulking surgery** Most effective *LPT vs. LPS* Depending on position, and size of lesion *Morcellation* It should be avoided, containment bags should be used **Hormonal treatment** It should be considered in case of patients unfit for surgery or as maintenance therapy after surgery	**Not-well defined** *TVUS and/or RMI* To evaluate regression, progression, or recurrence **Re-excision** It may be considered in case of residual lesions, or recurrences
US: ultrasound; TVUS: trans-vaginal ultrasound; MRI: magnetic resonance imaging; CT: computed tomography; PET: positron emission tomography; GISTs: gastrointestinal stromal tumors; LPT: laparotomy; LPS: laparoscopy.				

### 2.3. Benign metastasizing leiomyoma (BML)

BML is a rare disease of mesenchymal origin, characterized by the presence of single or multiple nodules composed of benign leiomyoma cells located outside the uterus. It can affect various sites, most commonly the lungs (approximately 80%) and lymph nodes. Other affected areas include the omentum, pelvis, abdomen, mediastinum, vertebrae, nervous system, bone, skeletal muscle, skin, heart (about 15 cases reported in the literature), breast, trachea, esophagus, and liver [38,39]. The first in the literature to describe this condition was Steiner in Ref. [40]. Approximately 150 cases have been described in the literature [41].

#### 2.3.1. Epidemiology

BML is usually found in premenopausal patients with a history of uterine leiomyomatosis who have undergone surgery (myomectomy or hysterectomy) [3]. Even though patients undergoing surgery are the most affected in the literature, no statistical significance has been shown towards one type of surgery over the others (laparotomy versus laparoscopy, hysterectomy versus myomectomy) [3]. The limitation may be the few cases described. In addition, the morcellation technique has only been adopted in the last 10 years, and this causes a lack of data in the long term. The average age at diagnosis varies from 47 to 54 years (range 34–69 years), and no risk factors such as ethnicity have been highlighted in literature [3].

#### 2.3.2. Pathogenesis

The pathogenesis of BML is not yet well defined; there are many theories about it. Among them, the most widely accepted is the spread of myometrial cells during operations involving the opening of the uterine cavity with vascular trauma and blood diffusion [8]. However, this does not provide an explanation for those cases of patients who have never undergone surgery. Another hypothesis is that of metastatic spread by blood and lymphatics from well-differentiated LMSs, which would support the metastatic potential of such lesions [8]. The third theory is based on the process of hormone-mediated metaplasia from mesenchymal cells of the coelomic epithelium. This theory would explain the multiple sites of occurrence of BML nodules even in patients who have not undergone uterine surgery or who do not have a history of uterine leiomyomatosis [3]. The theory that subserous uterine leiomyomas grow and invade surrounding areas only explains nodules close to the uterus and not the presence of nodules at distant sites [42].

#### 2.3.3. Diagnosis

Mostly, BML is an asymptomatic condition with an incidental instrumental diagnosis performed for other causes. When symptoms are present, they are related to the location of the nodules, particularly since pulmonary nodules are the most common; the most frequent symptoms, in more than 30% of cases, are respiratory (such as cough, dyspnea, chest pain), and rarely respiratory failure could occur [3,43]. However, in case of lymph node or cardiac involvement, these are more usually asymptomatic, while localized pain may be the manifestation of a bone implant [38]. Pulmonary embolism and hemothorax are rare manifestations of the disease, mostly linked to extensive lesions not treated surgically or pharmacologically [44].

Being a usually indolent condition, the time interval between uterine leiomyomatosis surgery and diagnosis approximately is 9 years, ranging from an early diagnosis shortly after surgery to up to 30 years after surgery [45]. Diagnosis is based on imaging techniques such as CT scans of the chest and abdomen with findings of multiple well-circumscribed nodules, most frequently bilateral, which may vary in size from a few millimeters to larger volumes. They are rarely calcific and may sometimes be described as cavitated nodules [8].

The definitive diagnosis is based on histological analysis of the lesion by biopsy [38]. A differential diagnosis should be made between lung carcinoma, hamartoma, lymphangioleiomyomatosis, Perivascular Epithelioid Cell tumor (PEComa), LMS, distant metastases, and infectious diseases. The patient’s medical history is essential for the differential diagnosis. Positron emission tomography (PET) with 18 F-fluorodeoxyglucose (FDG) is performed for differential diagnosis with malignant lesions; BML nodules have a mild uptake in contrast to LMS or metastases, which has a marked glycolytic metabolism, in a few cases of BML it is associated with a high uptake, but this does not seem to be related to the malignancy of the disease [46]. Histologically, nodules are composed of well-differentiated spindle cells structured in fascicles and express proteins typical of smooth muscle cells, such as smooth muscle actin (SMA), desmin, and caldesmon. As far as immunohistochemistry is concerned, they have a low proliferation rate (Ki67 < 5%) and do not usually present more typically malignant features such as necrosis, cell pleomorphism, or atypia. Frequently, ER and PR positivity is found, thus demonstrating a possible hormonal involvement in development/growth. Some chromosomal studies have shown 19q and 22q terminal deletions, unlike uterine myomas, and balanced translocations [3,38].

BML has an indolent course with a good prognosis. Only four cases are described in the literature with malignant transformation. More studies have been conducted to validate biomarkers to determine the malignant potential of the lesions, including the expression of certain microRNAs, such as miRNA-126 and miRNA-221, which could be useful to differentiate BML from LMS [47].

#### 2.3.4. Treatment

Treatment for BML is not standardised and should be modulated according to the site of interest and symptomatology. Despite the benign behavior and slow growth, surveillance by CT should be indicated for all patients. Those who present symptoms or progressing lesions should be offered treatments such as surgery or hormone therapy [48]. If there is no response to medical therapy or if compressive symptoms occur, surgical excision of the BML is indicated [43]. With regard to surgery, lung lesions can be approached by bronchoscopic excision or lung surgery. Nodules of BML respond to estrogen deprivation hormone therapy for which bilateral ovariectomies or medical hormone therapy such as GnRH agonists, SERMs such as tamoxifen, raloxifene, SPRM, and AI (anastrozole and letrozole) may be indicated. GnRH agonist therapy, other than suppressing the release of gonadotropins, acts by reducing the expression of Transforming growth factor beta (TGF-β3), a molecule involved in cell proliferation, which could lead to a volumetric reduction [8,38]. Progesterone therapy has shown more variable efficacy for BML treatment; it acts on the hypothalamic–pituitary axis by reducing gonadal estrogen release, however, in some cases, it appears to inhibit apoptosis of proliferative cells, causing an ineffective response [49]. Furthermore, the combination of GnRH agonists and raloxifene seems to have a positive effect in halting the evolution of BML and does not have an adverse effect on bone metabolism [50]. As far as chemotherapy is concerned, it does not seem to be effective in these patients as BML presents a continuous but slow growth with a low degree of mitosis [49].

#### 2.3.5. Prognosis and follow-up

The prognosis is favourable for patients with BML. A survival rate of 94 months has been calculated in the literature for surgically resected lung BML [51]. The close and prolonged follow-up of patients aims to diagnose any relapse or progression of the pathology early. However, there is a lack of shared protocols in managing this pathology [41].

The diagnostic approach, therapeutic options, and follow-up strategies for BML cases are outlined in Box 3.

Box 3. Diagnosis, treatment, and follow-up of benign metastatic leiomyoma (BML).

**Table:** 

**Benign metastatic leiomyoma**				
**Clinical presentations**	**Diagnostic tools**	**Differential diagnosis**	**Treatment modalities**	**Follow-up**
**Symptoms**(depending on sites) Dyspnea (most common) Abdominal/pelvic pain Asthenia Bone-related symptoms Compression of adjacent organs **Signs** (depending on sites) Pelvic mass Vaginal bleeding Abdominal distension **Asymptomatic** >1/3 of cases	**Chest X-ray** Pulmonary nodules are the most common; limited imaging quality **CT scan** To assess the localization, and sizes of lesions; particularly useful in case of pulmonary nodules **MRI scan** To better differentiate from malignancies assessing soft tissue characteristics **PET-CT/MRI** If malignancy is suspected **Radiologic guided-biopsy** It should be avoided due to low diagnostic accuracy **Histopathology** To obtain definitive diagnosis	Lung carcinoma Hamartoma LAM PEComa Leiomyosarcoma Distant metastases Infectious diseases	**Multidisciplinary discussion** To determine the best therapeutical approach, the potential surgical radicality, and patient’s operability **Surgical resection** In case of resectable lesions, symptomatic cases or growing lesions *Surgical access* Depending on position, numbers, and size of masses *Morcellation* It should be avoided, containment bags should be used **Hormonal treatment** It should be considered in case of patients unfit for surgery, unresectable lesions, or as maintenance therapy after surgery	**Not-well defined** *Diagnostic tools depending on sites* To evaluate regression, progression, or recurrence *CT scan* Preferred in case of pulmonary disease **Re-excision** It may be considered in case of residual lesions, or recurrences
CT: computed tomography; MRI: magnetic resonance imaging; PET: positron emission tomography; LAM: lymphangioleiomyomatosis; PEComa: perivascular epithelioid cell tumor.				

### 2.4. Intravenous leiomyomatosis (IVL)

IVL is a peculiar pathology characterized by the extension and invasion of histologically benign smooth muscle lesions within the pelvic and systemic vasculature. Intravenous leiomyoma may extend into the vascular spaces from the intrauterine to the systemic venules, including the common iliac vein and the inferior vena cava (IVC), without invading endothelial tissue; occasionally, it also involves the ovarian vein. If the lesion extends into the right heart chambers and pulmonary arteries, it is termed intracardiac leiomyomatosis [52].

#### 2.4.1. Epidemiology

Although uterine leiomyomatosis is a common condition, intravenous and intracardiac leiomyomatosis are very rare conditions, the first of which was described by Birsch–Hirschfeld in 1896. This condition accounts for approximately 0.25–0.40% of all uterine leiomyomas [43]. In fact, the main risk factors for developing this condition are a positive family history of uterine leiomyomatosis and previous gynecologic surgery (e.g. myomectomy or hysterectomy) [3].

#### 2.4.2. Pathogenesis

Because of the rarity of the condition, its etiology is still unclear. Two theories have been described. The first, developed by Steinmetz, postulates that IVL arises from a preexisting lipomatous uterine mass that grows and progressively invades the lumen of the vascular bed [53]. The second, developed by Knauer, describes IVL as a pathology resulting from a process of metaplasia of the smooth muscle cells of the uterine vascular wall [43].

In addition, gene dysregulation seems to be involved in the etiopathogenesis of IVL transformation, like the expression of the HMGA2 gene, which encodes for a transcription factor. HMGA2 is frequently associated with both malignant and benign tumorigenic processes in adult human tissues, as well as with certain characteristic mutations that promote the neoplastic process [54].

In studying the molecular profile of IVL, it was found that there are differences between the genetic signature of uterine leiomyomas and that of IVL, like elevated HOXA13 gene expression level, upregulated anti‐apoptosis‐related genes BCL2A1 and CDKN2A and the downregulated angiogenesis‐related gene CXCL8 [55]. In addition, a high rate of chromosomal aberration was observed in IVL [43].

#### 2.4.3. Diagnosis

Symptoms of IVL and its clinical manifestations depend on the location and the degree of involvement of the venous circulation. Patients could present with hemorrhagic menstrual cycles and abdominal pain but also with edema of the lower extremities (in patients with IVC involvement), chest pain, dyspnea, acute heart failure, and sudden death (in patients with cardiac involvement) [43].

Diagnosis could be problematic because symptoms only appear when there is direct involvement of the cardiovascular system. The most used diagnostic techniques for the diagnosis and follow-up of IVL are abdominal and pelvic ultrasonography, MRI, CT scan, and echocardiography [56]. CT scans seem to have a superior diagnostic ability to MRIs in terms of examining the extent of the tumor [57]. In case of cardiac involvement, echocardiography is the first-choice technique, which allows for the investigation of the presence of typically filiform masses resembling ‘walking stickheads’ or ‘snakeheads’ [52]. If the imaging shows the presence of a mass in the right atrium, originating from the IVC, the differential diagnosis should include metastatic tumors, thrombi, cardiac myxomas, primary LMS and lymphoma. Furthermore, when a patient with IVL presents with pulmonary nodules, it is important to consider the possible diagnosis of BML [52].

#### 2.4.4. Classification

Determining the extent of the tumor is very important for planning the type of management and, thus, the type of treatment. The Mayo classification is used for tumor staging at the level of the vena cava. There are four levels: I, the tumor is 2 cm from the confluence of the renal vein and the IVC; II: extends 2 cm above the confluence of the renal vein and the IVC but remains below the hepatic veins; III: involves the intrahepatic IVC; IV: extends above the diaphragm or into the right atrium [58]. A further classification has been proposed for IVL, called the ‘The Anzhen quaternary classification’: first grade refers to the size of the intracardiac portion relative to the diameter of the IVC (type N and Y); second grade refers to the size of the intravenous portion relative to the diameter of the IVC (type A, B, C, D); third grade refers to the area of origin (type I originated from the internal iliac vein; type O from ovarian vein); fourth grade refers to the laterality of the lesion in the pelvis (type L or R) [59].

#### 2.4.5. Treatment

Treatment depends on various factors (location, size, age of the patient) but should always aim at complete resection of the tumor. The most effective treatment is based on a multidisciplinary surgical approach with a single-stage or two-stage procedure. In most cases, the single-stage approach is used, which simultaneously exposes the abdominal and thoracic cavities, with excellent success both in terms of resolving the pathology and reducing complications. However, as it is not always possible to use the latter, in some cases, it will be necessary to operate with the two-stage procedure; thus, the cardiac leiomyomas are removed first, and then the pelvic leiomyomas will be removed by means of hysterectomy combined with ovariectomy [60].

Cardiopulmonary by-pass (CPB) is not always mandatory; however, it is mainly used in cases where the tumor is large or firmly adheres to cardiac or venous structures; the procedure without CPB can be performed if the tumor is small and the blood flow around the IVL is regular [60].

Other useful techniques in association with surgery are intraoperative transesophageal echocardiographic monitoring, mobilization of the liver and its rotation on the left side of the abdomen, and implantation of double-J stents on the ureters [60].

The use of hormonal therapies in combination with surgery is still controversial. It has been seen that the administration of GnRH agonists before surgery reduces the size of the myoma, increasing the success of surgery. In addition, the administration of GnRH agonists after surgery prevents the risk of recurrence. Other hormonal therapies include SERMs and AIs, which are used both as monotherapy and as adjuvant treatments for IVL [55]. Patients with a desire to preserve fertility may undergo simple myomectomy. The choice of surgical technique (laparoscopic versus laparotomic) appears to have no impact on the recurrence rate, depending on the surgeon, the size of the tumor and the patient’s condition [60].

#### 2.4.6. Prognosis and follow-up

Recurrence of these tumors is high, at a rate of 30%, with a better outcome in patients undergoing total hysterectomy and bilateral ovariectomy [55,58–60]. Long-term follow-up is necessary, particularly in patients undergoing incomplete tumor resection with preservation of the uterus and ovaries. Although there are no standard recommendations, follow-up every 3–6 months for the first 2 years after surgery and then every 12 months is suggested [55].

Box 4 presents an overview of the diagnosis, treatment, and monitoring of IVL cases.

Box 4. Diagnosis, treatment, and follow-up of intravascular leiomyoma (IVL).

**Table:** 

**Intravascular leiomyoma**				
**Clinical presentations**	**Diagnostic tools**	**Differential diagnosis**	**Treatment modalities**	**Follow-up**
**Symptoms**(depending on sites, and sizes) Dyspnea (most common) Abdominal/pelvic pain Thrombosis-related symptoms **Signs** (depending on sites, and sizes) Pelvic mass Vaginal bleeding Abdominal distension **Asymptomatic** < 1/3 of cases	**Doppler US and/or TVUS** To initially detect intravascular lesions, to assess any abnormality of blood flow, but low accuracy in mass characterization **Echocardiography** Useful in case of cardiac involvement **CT angiography** To map vascular involvement **MRI with contrast** To better characterize the soft tissues of lesions **PET-CT/MRI** If malignancy is suspected **Histopathology** To obtain definitive diagnosis	Leiomyosarcoma Intravenous thrombus PEComa Metastatic disease	**Multidisciplinary discussion** To determine the best therapeutical approach, the potential surgical radicality, and patient’s operability **Surgical resection** Preferred treatment to achieve complete surgical removal *Surgical access* Depending on position, and size of lesion *Morcellation* It should be avoided, containment bags should be used *CPB* Useful in case of lesions strictly adherent to cardiac structures **Hormonal treatment** It should be avoided as primary treatment due to high risk of cardiovascular events; useful as maintenance therapy after surgery	**Not-well defined** *Echocardiography and/or Doppler US* To evaluate regression, progression, or recurrence **Re-excision** It may be considered in case of residual lesions, or recurrences
US: ultrasound; TVUS: trans-vaginal ultrasound; CT: computed tomography; MRI: magnetic resonance imaging; PET: positron emission tomography; PEComa: perivascular epithelioid cell tumor; CBP: cardiopulmonary by-pass.				

## 3. Materials and methods

This systematic review was conducted in accordance with the PRISMA (Preferred Reporting Item for Systematic Reviews and Meta-Analyses) statement, as delineated in the PRISMA checklist [61], and registered with PROSPERO (CRD42024496523; https://www.crd.york.ac.uk/prospero accessed on 22 March 2025). The study adhered to the following Population, Intervention, Comparison, Outcome (PICO) questions:

- *Population*: patients diagnosed with one of the ESLs (DPL, parasitic myoma, BML, or IVL);
- *Intervention*: any type of surgery (gynaecological and non-gynaecological) or hormonal treatment performed for uterine mass or related symptoms;
- *Comparison*: different histopathological and clinical features of patients within each different category of ESL;
- *Outcomes*: (1) Description of the clinical and histopathological features of each different category of ESL; (2) Clinical outcomes of patients undergoing medical or surgical treatment for ESL; (3) Comparison of surgical data between intra- and extra-abdominal diseases.

### 3.1. Search and selection

The search was conducted electronically on three bibliographic databases, including Pubmed, Scopus, and Web of Science on 17 January 2024, without any filters applied. Search terms used were related to the type of lesions: (1) leiomyomatosis peritonealis disseminata/disseminated peritoneal leiomyomatosis, (2) parasitic myoma, (3) metastasizing leiomyoma/benign metastatic leiomyoma, and (4) intravascular leiomyoma/intravenous leiomyoma. The nomenclature used for the classification of the lesions reported above were in accordance with WHO/IARC Classification of Female genital tumours published in 2020 [62].

The search strategy observed the following systematic approach, starting from the Pubmed database:

#1 search: first search term (OR synonyms, OR plurals, OR different forms, OR abbreviations) [text word];

#2 search: first search term [MeSH Terms];

#3 search: combine with ‘AND’ each string on synonyms and alternative terms; combine with ‘OR’ each string on. The complete search strategy employed in this systematic review is available in the Supplementary File S1 (Tables S1–S3).

Duplicate records retrieved from the online database search were identified and removed using an Excel spreadsheet based on Digital Object Identifier (DOI) matching. Thereafter, two independent reviewers (J.D.G., L.N.) selected the studies using a two-step screening method. At first, the screening of titles and abstracts was performed to assess eligibility. Only case reports and case series written in English language addressing the diseases of interest were included, while abstracts, cost effectiveness studies, letters to the editor, conference proceedings, cross-sectional studies, cross-sectional case control studies, all type of review articles, and meta-analyses were excluded. Studies uncompleted, retracted, conducted on animal, and those not relevant to the topic of interest were also omitted.

As a second step, the two reviewers assessed the full-texts of included articles to exclude those lacking comprehensive descriptions of cases including clinical, surgical, and histological data. The same authors manually searched reference lists to find for additional relevant publications. Any disagreements between individual judgements were resolved by a third reviewer (A.C.).

### 3.2. Data extraction

Data collection from all included studies was carried out by five independent authors [A.A., M.B., D.C., C.G., L.G. (Linda Grementieri) and J.P.] using a pre-formed spreadsheet in Microsoft Excel. A seventh author (A.C.) performed an independent data extraction for verification purposes. The following details were gathered from each study:

- *General information*: title, first author, DOI, year of publication, study design;
- *Demographic and clinical characteristics*: age, parity, race, body mass index (BMI), main symptom, main sign, presence/absence of anemia, type of ESL, number of masses described, maximum dimensions of lesions described, first diagnostic tool applied;
- *Treatment data*: surgical (age of patients, CA125 measurement, surgical access, type of surgery, morcellation) and medical treatment data;
- *Histopathological data*: presence of mitosis, atypia, necrosis; type of leiomyoma variants; desmin, H-caldesmone, Smooth Muscle Actin (SMA), Muscle Specific Actin (MSA), calponine, Smooth Muscle Myosin Heavy Chain (SMMHC), Estrogen Receptor (ER), Progesterone Receptor (PR), Wilms Tumor 1 (WT1), Oxytocin receptor positivity or negativity.
- *Outcome data*: collected as reduction or resolution of clinical presentation, decrease in mass size, or unfavorable outcome.

The classification of leiomyoma variants used was in accordance with WHO/IARC Classification of Female genital tumours published in 2020 [62]. To classify the race of patients, the categorization developed by United States Office of Management and Budget (OMB) in 1997 was followed [63].

### 3.3. Data synthesis and analysis

The collected data were reported as continuous or categorical variables. Continuous variables were tested for normal or not-normal distribution using the D’Agostino Pearson test. According to distribution, they were expressed as mean standard deviation or median and range. Categorical variables were expressed as frequency and percentage, and were compared with Chi-square or Fisher’s exact test. A *p* value of less than 0.05 was considered statistically significant.

Finally, the cases of DPL, and PM were grouped in the Intra-Abdominal Extrauterine Leiomyoma (IAEL) category, while the cases of BML, and IVL were included in the Extra-Abdominal Extrauterine Leiomyoma (EAEL) group for comparison of surgical data. A comparison of the type of treatment (demolition or conservative) before the diagnosis of ESLs between these two groups was performed. MedCalc^®^ Statistical Software version 20 (MedCalc Software Ltd., Ostend, Belgium) was used for statistical analysis.

### 3.4. Assessment of risk of bias

Two authors [G.D.C. and L.G. (Luca Giannella)] conducted the quality assessment of the included studies independently, utilizing the Joanna Briggs Institute (JBI) critical appraisal checklist for case reports and case series [64,65]. Any disagreements between them were addressed and resolved through discussion with a third author (A.C.).

The results of quality assessment of included case reports, and case series are available in Supplementary File S2 (Figures S1 and S^2^).

**Figure 1.**
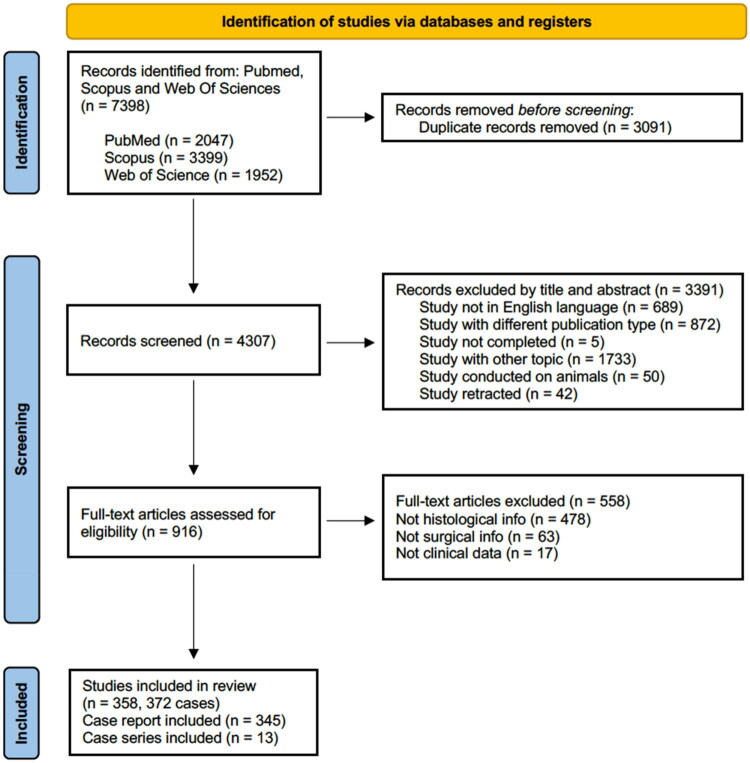
Literature review flow chart.

## 4. Results

### 4.1. Literature review details

We retrieved 2047 articles from the PubMed database, 3399 from Scopus, and 1952 from Web of Science (accessed on 17 January 2024); then, 3091 duplicate records were removed. Based on the title and abstract, 3391 records were excluded. Next, the full text of 916 papers was assessed for eligibility. Following the inclusion and exclusion criteria, 558 articles were further excluded. Finally, 358 studies were evaluated for qualitative synthesis, including 372 cases (Figure 1). All patients met the inclusion criteria, and clinical, histological, and treatment data were collected. Details of all studies and patient data collected but not reported in the study can be found in Supplementary File S1 (Tables S4–S^7^).

### 4.2. Patient characteristics

Table 1 presents a comparative analysis of patient characteristics across the four groups, considering the IAELs, and EAELs.

**Table 1.** Patient characteristics diagnosed with ESLs.

	DPL	PM	IAEL	BML	IVL	EAEL
Characteristics	*n* (%)	*n* (%)	*n* (%)	*n* (%)	*n* (%)	*n* (%)
**Age** (years) (median, range)	42 (18–78)	41.5 (21–63)		46 (26–77)	47 (25–77)	
**Parity**	**62 cases**	**22 cases**	**84 cases**	**45 cases**	**47 cases**	**92 cases**
Nulliparous	30 (48.4)	3 (13.6)	33 (39.4)	9 (20.0)	11 (23.4)	20 (21.7)
Multiparous	32 (51.6)	19 (86.4)	51 (60.6)	36 (80.0)	36 (76.6)	72 (78.3)
**Symptom (predominant)**	**86 cases**	**26 cases**	**112 cases**	**138 cases**	**87 cases**	**225 cases**
Dysmenorrhea	0 (–)	2 (7.7)	2 (1.8)	0 (–)	0 (–)	0 (–)
Abdominal/pelvic pain	55 (63.9)	18 (69.2)	73 (66.4)	22 (15.9)	23 (26.4)	45 (20.0)
Compression of adjacent organs	5 (5.8)	3 (11.5)	8 (7.3)	6 (4.4)	5 (5.7)	11 (4.9)
Asthenia	0 (–)	1 (3.9)	1 (0.9)	4 (2.9)	1 (1.2)	5 (2.2)
Dyspnea	1 (1.2)	0 (–)	1 (0.9)	41 (29.7)	26 (29.9)	67 (29.8)
Thrombosis-related	0 (–)	0 (–)	0 (–)	4 (2.9)	13 (14.9)	17 (7.6)
Bone-related	1 (1.2)	0 (–)	1 (0.9)	7 (5.1)	0 (–)	7 (3.1)
No symptoms	24 (27.9)	2 (7.7)	26 (21.8)	54 (39.1)	19 (21.9)	73 (32.4)
**Sign (predominant)**	**71 cases**	**29 cases**	**100 cases**	**111 cases**	**71 cases**	**182 cases**
Pelvic mass	27 (38.0)	13 (44.8)	40 (40.0)	19 (17.1)	13 (18.3)	32 (17.6)
Vaginal bleeding	7 (9.9)	6 (20.7)	13 (13.0)	12 (10.8)	10 (14.1)	22 (12.1)
Abdominal distension	20 (28.2)	3 (10.4)	23 (23.0)	8 (7.2)	8 (11.3)	16 (8.8)
No signs	17 (23.9)	7 (24.1)	24 (24.0)	72 (64.9)	40 (56.3)	112 (61.5)
**Surgical time interval**^a^ (months) (median, range)	48 (0–252)	120 (12–240)		96 (0–372)	48 (0–684)	
**Previous surgery** ^b^	**93 cases**	**30 cases**	**123 cases**	**155 cases**	**94 cases**	**249 cases**
None	25 (26.9)	7 (23.3)	32 (26.0)	15 (9.7)	37 (39.4)	52 (20.9)
Myomectomy	31 (33.3)	5 (16.7)	36 (29.3)	43 (27.8)	14 (14.9)	57 (22.9)
Hysterectomy	28 (30.1)	8 (26.7)	36 (29.3)	82 (52.9)	32 (34.0)	114 (45.8)
SH	1 (1.1)	1 (3.3)	2 (1.6)	9 (5.8)	2 (2.1)	11 (4.4)
Cesarean section	4 (4.3)	4 (13.3)	8 (6.5)	5 (3.2)	5 (5.3)	10 (4.0)
Non-gynaecological surgery	4 (4.3)	5 (16.7)	9 (7.3)	1 (0.6)	4 (4.3)	5 (2.0)
**Morcellation**	**17 cases**	**4 cases**	**21 cases**	**34 cases**	**13 cases**	**47 cases**
Yes	12 (70.6)	4 (100.0)	16 (76.2)	0 (–)	1 (7.7)	1 (2.1)
No or in a bag	5 (29.4)	0 (–)	5 (23.8)	34 (100.0)	12 (92.3)	46 (97.9)

Only cases with available data are reported.

DPL: disseminated peritoneal leiomyomatosis; PM: parasitic myoma; IAEL: intra-abdominal extrauterine leiomyomas (DPL + PM);BML: benign metastatic leiomyoma; IVL: intravascular leiomyoma; EAEL: extra-abdominal extrauterine leiomyomas (BML + IVL); SH: supracervical hysterectomy.

^a^ Months between the first and second surgery (treatment of ESLs).

^b^ Previous surgery before diagnosis of ESLs.

The median age at diagnosis ranges from 41.5 years in the PM group to 47 years in the IVL group, showing a higher age in patients with EAELs compared to those with IAELs, suggesting a possible later clinical manifestation in the former. Parity analysis reveals that multiparous patients constitute the majority in all groups, with the highest proportion observed in the PM group (86.4%), while nulliparity is more common in DPL (48.4%).

BMI shows notable differences: lower BMI (<30 kg/m^2^) is predominant in DPL (71.4%), whereas higher BMI categories (≥30 kg/m^2^) are more frequent in BML and IVL, suggesting a possible association with obesity-related factors. Racial distribution highlights a higher proportion of Asian patients across all groups, ranging from 37.5% to 45.5%, while White individuals also represent a considerable portion, particularly in DPL and PM groups.

Abdominal or pelvic pain is the most frequently reported symptom, especially among IAELs patients (63.9% in DPL, 69.2% in PMs, and 61.8% overall). Conversely, dyspnea is almost exclusively associated with IVL (29.9%), likely due to its vascular involvement. Thrombosis-related symptoms are also particular to IVL (14.9%) and absent in IAELs. Notably, 73,7% of EAELs are completely asymptomatic, a significantly higher percentage than IAELs.

Regarding clinical signs, pelvic mass is observed in 38.0% of DPL cases and 44.8% of PMs cases, indicating a common feature of IAELs.

A significant proportion of patients have a history of previous gynecological surgery, with myomectomy and hysterectomy being the most common interventions. Prior myomectomy is particularly frequent in IAELs (38.71%), while hysterectomy is more common in EAELs (76.0%).

The previous surgical approach varies: laparoscopic myomectomy is highly prevalent in IAELs (86.36%), whereas laparotomic hysterectomy is more frequent in EAELs (70.7%). Interestingly, morcellation is performed in 94,1% of cases before the diagnosis of IAELs, suggesting the potential role of morcellation in disseminating intra-abdominal lesions.

The median interval between the first and second surgery (after diagnosis) varies significantly, ranging from 48 months in IAELs to 96 months in EAELs. This long latency suggests that specific leiomyomatosis subtypes may remain indolent for extended periods before requiring further surgical intervention.

Data on BMI, race, and type of previous surgery are available in Supplementary File S2 (Table S8).

### 4.3. Lesion characteristics

Table 2 provides a comparative overview of lesion characteristics. The median number of masses described varies slightly, highest in DPL, and BML (2, with ranges of 1–12 and 1–30, respectively) and lower in PM, and IVL (1, ranges of 1–3 and 1–4, respectively). Notably, PMs has the largest individual masses, with a median maximum dimension of 10.35 cm (range 2–43 cm), while BML had generally smaller masses (median 4 cm). EAELs, particularly IVL, also have a sizeable maximum dimension (median 10 cm, range 1–47 cm), suggesting a propensity for more extensive growth in this subgroup.

**Table 2.** ESLs characteristics.

	DPL	PM	IAEL	BML	IVL	EAEL
Characteristics	*n* (%)	*n* (%)	*n* (%)	*n* (%)	*n* (%)	*n* (%)
**No. of masses** (median, range)	2 (1–12)	1 (1–3)		2 (1–30)	1 (1–4)	
**Max dimension**^a^ (median, range)	7 (0.5–30)	10.35 (2–43)		4 (1–30)	10 (1–47)	
**Diagnostic tools** ^b^	**89 cases**	**30 cases**	**119 cases**	**152 cases**	**91 cases**	**243 cases**
US	32 (36.0)	9 (30.0)	41 (34.5)	15 (9.9)	39 (42.9)	54 (22.2)
MR	6 (6.7)	11 (36.6)	17 (14.3)	16 (10.5)	7 (7.7)	23 (9.5)
CT	30 (33.7)	8 (26.6)	38 (31.9)	78 (51.3)	37 (40.7)	115 (47.3)
Surgery	21 (23.6)	1 (3.4)	22 (18.5)	1 (0.7)	2 (2.3)	3 (1.2)
Others	0 (–)	1 (3.4)	1 (0.8)	42 (27.6)	6 (6.6)	48 (19.8)
**Mitosis**	**77 cases**	**23 cases**	**100 cases**	**123 cases**	**75 cases**	**198 cases**
0–4	65 (84.4)	22 (95.7)	87 (87.0)	115 (93.5)	70 (93.3)	185 (93.4)
5–9	8 (10.4)	0 (–)	8 (8.0)	7 (5.7)	4 (5.4)	11 (5.6)
≥10	4 (5.2)	1 (4.3)	5 (5.0)	1 (0.8)	1 (1.3)	2 (1.0)
**Atypia**	**75 cases**	**19 cases**	**94 cases**	**111 cases**	**74 cases**	**185 cases**
None	57 (76.0)	18 (94.7)	75 (79.8)	91 (82.0)	63 (85.1)	154 (83.2)
Mild	14 (18.7)	1 (5.3)	15 (16.0)	19 (17.1)	8 (10.8)	27 (14.6)
Moderate	1 (1.3)	0 (–)	1 (1.1)	0 (–)	2 (2.7)	2 (1.1)
Severe	3 (4.0)	0 (–)	3 (3.2)	1 (0.9)	1 (1.4)	2 (1.1)
**Necrosis**	**54 cases**	**20 cases**	**74 cases**	**99 cases**	**48 cases**	**147 cases**
Absent	46 (85.2)	14 (70.0)	60 (81.1)	89 (89.9)	41 (85.4)	130 (88.4)
Present	8 (14.8)	6 (30.0)	14 (18.9)	10 (10.1)	7 (14.6)	17 (11.6)

Only cases with available data are reported.

DPL: disseminated peritoneal leiomyomatosis;PM: parasitic myoma; IAEL: intra-abdominal extrauterine leiomyomas (DPL + PM); BML: benign metastatic leiomyoma; IVL: intravascular leiomyoma; EAEL: extra-abdominal extrauterine leiomyomas (BML + IVL); US: ultrasound; MR: magnetic resonance; CT: computed tomography.

^a^ Max dimension of masses described.

^b^ Diagnostic tools which firstly lead to diagnosis.

US is the most frequently used imaging tool in IAELs, especially in DPL (36%) and BML (43.2%). In contrast, it is used less frequently in EAELs (9.9% in IVL, 42.9% in BML). MRI was used in 36.6% of PM cases but is less commonly applied to other subgroups. CT scanning is the primary diagnostic tool in EAELs (51.3% in IVL and 40.7% in BML), higher than IAELs (24.8%), indicating the importance of CT in detecting extra-abdominal manifestations. Surgical diagnosis is rare in EAELs but more frequent in IAELs (23.6% in DPL), underscoring different diagnostic approaches based on lesion location.

Mitotic rate analysis reveals that most lesions exhibit low mitotic activity [0–4 mitoses per 10 high-power field (HPF)], particularly in IVL (93.3%) and IAELs (84.4% in DPL and 95.7% in PM). However, higher mitotic activity (≥10 per 10 HPF) is more frequent in DPL and PM lesions, suggesting more aggressive growth potential in a subset of IAELs. Atypia is predominantly absent or mild, with severe atypia being rare across all groups. Necrosis is generally absent, though a higher proportion has been noted in PM lesions (30%). Desmin expression was consistently high across all groups, exceeding 94%. Other biomarkers like SMA, ER, and PR are mostly positive, highlighting consistency in smooth muscle origin across the groups.

Data on immunohistochemistry after the surgery performed before diagnosis of ESLs, and immunohistochemistry after surgery for ESLs are available in Supplementary File S2 (Tables S9 and S^10^).

### 4.4. Treatment and outcomes

Table 3 outlines the management strategies and outcomes for the four groups. Surgery alone is the most common treatment across all groups, with the highest prevalence in PM (86.7%) and IVL (80.8%), followed by DPL (76.3%) and BML (52.2%). Notably, a combined surgery and medical treatment approach is more commonly used in BML (37.4%) than in other subtypes, suggesting a greater reliance on adjuvant therapy for managing this condition. Medical treatment alone is rare (≤5.4% in all subtypes), with IVL patients having the lowest frequency (1.1%), indicating that non-surgical management is generally not the preferred approach. Follow-up alone is only used in a small percentage of BML (5.2%) and IVL (1.1%) cases, while no IAELs are managed with observation alone.

**Table 3.** Management strategies and outcomes in patients with ESLs.

	DPL	PM	IAEL	BML	IVL	EAEL
Characteristics	*n* (%)	*n* (%)	*n* (%)	*n* (%)	*n* (%)	*n* (%)
**Management**	**93 cases**	**30 cases**	**123 cases**	**155 cases**	**94 cases**	**249 cases**
Surgery alone	71 (76.3)	26 (86.7)	97 (78.8)	81 (52.2)	76 (80.8)	157 (63.1)
MT alone	5 (5.4)	0 (–)	5 (4.1)	8 (5.2)	1 (1.1)	9 (3.6)
Surgery + MT	17 (18.3)	4 (13.3)	21 (17.1)	58 (37.4)	16 (17.0)	74 (29.7)
Follow-up alone	0 (–)	0 (–)	0 (–)	8 (5.2)	1 (1.1)	9 (3.6)
**Surgical access**	**81 cases**	**30 cases**	**111 cases**	**123 cases**	**83 cases**	**206 cases**
Laparoscopic	15 (18.6)	5 (16.7)	20 (18.0)	5 (4.1)	0 (–)	5 (2.4)
Laparotomic	62 (76.5)	21 (70.0)	83 (74.8)	36 (29.4)	54 (65.1)	90 (43.7)
Hysteroscopic	0 (–)	0 (–)	0 (–)	0 (–)	0 (–)	0 (–)
Others	4 (4.9)	4 (13.3)	8 (7.2)	82 (66.7)	29 (34.9)	111 (53.9)
**Medical treatment**	**93 cases**	**30 cases**	**123 cases**	**155 cases**	**94 cases**	**249 cases**
No	71 (76.3)	26 (86.7)	97 (78.9)	89 (57.4)	77 (81.9)	166 (66.7)
Mifepristone	0 (–)	0 (–)	0 (–)	0 (–)	0 (–)	0 (–)
Ulipristal acetate	1 (1.1)	0 (–)	1 (0.8)	3 (1.9)	0 (–)	3 (1.2)
Analogues GnRH	8 (8.6)	3 (10.0)	11 (8.9)	33 (21.3)	8 (8.5)	41(16.5)
Others	13 (14.0)	1 (3.3)	14 (11.4)	30 (19.4)	9 (9.6)	39 (15.7)
**Outcomes**	**67 cases**	**24 cases**	**91 cases**	**127 cases**	**77 cases**	**204 cases**
Death	5 (7.5)	0 (–)	5 (5.5)	4 (3.1)	2 (2.6)	6 (2.9)
Less symptoms	3 (4.5)	2 (8.3)	5 (5.5)	9 (7.1)	3 (3.9)	12 (5.8)
No symptoms	39 (58.2)	21 (87.5)	60 (65.9)	48 (37.8)	59 (76.6)	107 (52.5)
Size mass decreased	11 (16.4)	0 (–)	11 (12.1)	20 (15.7)	1 (1.3)	21 (10.3)
No symptoms and size mass decreased	8 (11.9)	0 (–)	8 (8.8)	35 (27.6)	10 (13.0)	45 (22.1)
Size mass decreased and less symptoms	1 (1.5)	1 (4.2)	2 (2.2)	11 (8.7)	2 (2.6)	13 (6.4)

Only cases with available data are reported.

DPL: disseminated peritoneal leiomyomatosis; PM: parasitic myoma; IAEL: intra-abdominal extrauterine leiomyomas (DPL + PM); BML: benign metastatic leiomyoma; IVL: intravascular leiomyoma; EAEL: extra-abdominal extrauterine leiomyomas (BML + IVL); MT: medical treatment;GnRH: gonadotropin releasing hormone.

The surgical access method varied considerably. Laparotomy is the most frequent approach, while laparoscopy is rarely performed, with the highest frequency in DPL (18.6%), and PM (16.7%), but only in 4.1% of BML cases and not in IVL cases. Alternatives to gynecological surgery are highly prevalent in EAELs, suggesting the need for more specialized surgical interventions in these cases, possibly due to vascular involvement and metastatic potential.

A significant proportion of patients do not receive any medical treatment. The use of GnRH analogues is highest in BML (21.3%) and lower in IAELs, suggesting that hormonal therapy is more commonly employed in BML cases. Ulipristal acetate is used in a minimal number of cases (≤1.9%), and mifepristone is not used in any subgroup.

The analysis of patient outcomes reveals notable differences in symptom resolution and tumor progression among subtypes. Mortality is low across all subgroups (3.7%), with no deaths recorded in PMs. The highest mortality rate is observed in DPL.

Complete resolution of symptoms is most common in PM, and IVL (76.6%), while only 37.8% of BML and 58.2% of DPL cases achieve complete symptomatic relief. A combination of symptom relief and mass size reduction is more frequently observed in BML, supporting the hypothesis that this subgroup is more responsive to therapy than other subtypes.

### 4.5. Treatment performed before the diagnosis of ESL

Table 4 compares the types of treatment (demolition or conservative) performed before the diagnosis of ESLs. Among patients with IAELs, the treatments are nearly evenly split (51.3% hysterectomy vs. 48.7% myomectomy). Conversely, hysterectomy is more prevalent in EAELs, accounting for 68.7%, compared to 31.3% for myomectomy. The trend is even more pronounced for IVL, where hysterectomy constituted 70.8% of treatments performed before diagnosis. The Chi-square test was applied to assess the association between the type of uterine surgery (hysterectomy or myomectomy) and the localization of leiomyomatosis (IAELs, and EAELs). The analysis revealed a significant association (*p* = 0.042). Tables comparing the distribution of collected data for the main variables between IAELs and EAELs are available in Supplementary File S2 (Tables S11–S^13^).

**Table 4.** Comparison between individuals undergone hysterectomy/SH versus myomectomy as previous surgery performed for ESLs.

	DPL	PM	IAEL	BML	IVL	EAEL	
Characteristics	*n* (%)	*n* (%)	*n* (%)	*n* (%)	*n* (%)	*n* (%)	*p* value
	60 cases	14 cases	74 cases	134 cases	48 cases	182 cases	
**Hysterectomy/SH**	29 (48.3)	9 (64.3)	38 (51.3)	91 (67.9)	34 (70.8)	125 (68.7)	0.042^a^
**Myomectomy**	31 (51.7)	5 (35.7)	36 (48.7)	43 (32.1)	14 (29.2)	57 (31.3)	

Only cases with available data are reported.

DPL: disseminated peritoneal leiomyomatosis; PM: parasitic myoma; IAEL: intra-abdominal extrauterine leiomyomas (DPL + PM); BML: benign metastatic leiomyoma; IVL: intravascular leiomyoma; EAEL: extra-abdominal extrauterine leiomyomas (BML + IVL); SH: supracervical hysterectomy.

^a^ The *p* value was calculated using the Chi-square test comparing the data in the IAEL and EAEL columns.

## 5. Discussion

This review provides a detailed analysis of ESLs after performing a systematic literature search and data collection of all published cases of DPL, PM, BML, and IVL based on the inclusion and exclusion criteria. Although rare, these conditions determine significant diagnostic and therapeutic challenges due to their distinct pathogenesis, clinical presentation, and management strategies.

A key finding of this review is the significant variability in clinical presentation. IAELs often present with abdominal or pelvic pain, mass effect symptoms, or are discovered incidentally during surgery. In contrast, EAELs are often asymptomatic and often discovered incidentally on imaging. IVL, particularly when extending into the right atrium, can cause life-threatening cardiovascular symptoms such as heart failure and syncope [43]. The long latency period between primary surgery and symptom onset of ESL, ranging from months to decades, makes timely diagnosis difficult.

This review emphasizes the necessity of accurate characterization and personalized therapeutic approaches for ESL. Imaging modalities play an important role in distinguishing these tumors from malignancies like LMS. While US and MRI are preferred for intra-abdominal lesions, CT remains the gold standard for identifying pulmonary and vascular involvement in BML and IVL. Although helpful in differentiating benign from malignant lesions, PET-CT has shown variable results in BML cases, requiring histopathological confirmation [46]. Treatment approaches vary according to tumor location, severity of symptoms, and the patient’s reproductive status. Surgery remains the cornerstone of treatment, with complete tumor excision being the preferred strategy in symptomatic cases. Laparoscopic resection is feasible for IAELs, but extra-abdominal disease often requires laparotomy. In IVL cases with cardiac involvement, a multidisciplinary approach involving cardiothoracic and gynecological surgeons is essential to ensure the complete removal of tumor thrombi.

Medical therapy has an adjuvant role, particularly in hormone-sensitive tumors. GnRH analogs, AIs, and SERMs are effective in disease stabilization and symptom management. However, their effectiveness in the long term to avoid recurrence is doubtful. Notably, BML is more sensitive to medical treatment than the other subtypes, a finding that corroborates the theory of hormonal modulation on tumor growth [8,38].

Recurrence rates are extremely variable, with highest risk being seen in IVL, where persistence of disease can occur with incomplete resection of intravenous extensions. TAH and BSO decrease recurrence risk, particularly in hormone-sensitive disease [55,58–60]. Instances of recurrent disease despite ovaries removal nonetheless underscore the need for ongoing surveillance. Long-term follow-up with imaging remains essential, but standardized protocols for surveillance have yet to be established.

Our collected data suggest that previous surgical procedures such as hysterectomy involving ligation and cutting of major uterine vessels could be more implicated in the formation of EAELs, especially intravascular, like IVL or parenchymal metastases, like BML. Vascular integrity disruption during hysterectomy could facilitate hematogenous or lymphatic dissemination of smooth muscle cells with a greater possibility of extra-abdominal implantation. In contrast, myomectomy, especially laparoscopic myomectomy with morcellation, tends to lead to intraperitoneal metastatic spread predominantly as disseminated leiomyoma fragments implant in the peritoneal cavity. These findings imply that the selection of the surgical method is a significant factor influencing the route of disease dissemination and highlight the necessity for proper surgical methods and precautions to minimize the risk of tumor spread after surgery. Avoiding uncontrolled morcellation at the time of gynecologic surgery is a significant precaution. Containment bags and thorough peritoneal lavage can also minimize the danger of iatrogenic dissemination.

Furthermore, progress in molecular profiling can likewise create opportunities for targeted treatments with potential non-surgical management of recurrent or unresectable illnesses. Long-term outcomes, the impact of hormonal therapies on disease progression, and the genetic foundation of such conditions are all worthy of additional investigation. More extensive series of patients in multicenter studies are needed to help refine diagnostic approaches and create guidelines for best management.

### 5.1. Strengths and limitations

To our knowledge, this work represents the first systematic review to examine the available clinical literature on different types of ESLs, collecting data from case reports, and case series. The review was conducted according to a rigorous process. Each case report or case series was meticulously assessed by a minimum of two independent reviewers. A detailed analysis of published studies was performed, with a rigorous assessment of their quality.

We acknowledge that this systematic review is not without its limitations: (1) the literature on ESLs is limited, which is why only case reports, and case series were included; (2) the latter, by their very nature, exhibit limited methodological quality and consequently a high risk of bias; (3) despite meticulous selection, and data collection processes, some information was not available for all categories analysed, causing inevitable imbalances in the datasets; (4) the decision not to filter by publication year was taken in order to reduce the risk of selection bias. However, it should be considered that the management of these conditions may vary over time due to ongoing advances in both surgical, and medical therapies.

Due to these limitations, actually it is not possible to propose changes in surgical management of primary uterine leiomyomatosis.

## 6. Conclusions

DPL, PM, BML, and IVL represent a spectrum of ESLs with overlapping but distinct clinical and pathological features. While surgical excision remains the mainstay of treatment, a multimodal approach including imaging, histopathology, and surgical and medical management is critical for optimal patient outcomes. Increased awareness of these conditions among clinicians and advancement in diagnostic techniques, and molecular investigations will enhance patient care and guide future therapeutic developments.

## Supplementary Material

Supplementary File S2 ESL ANNALS.docx

Supplementary File S1 ESL ANNALS.docx

## Data Availability

The studies included in the systematic review were all searchable in the databases mentioned in the study. Data collected are available within the article and its supplementary files. Further details are available from the corresponding author upon reasonable request.

## Abbreviations

AI: Aromatase inhibitor
BMI: Body mass index
BML: Benign metastatic leiomyoma
BSO: Bilateral salpingo-oophorectomy
CA: Cancer antigen
CBP: Cardiopulmonary by-pass
CT: Computed tomography
DPL: Disseminated peritoneal leiomyomatosis
EAEL: Extra-abdominal extrauterine leiomyoma
ER: Estrogen receptor
ESL: Extrauterine sites of leiomyomatosis
FIGO: International Federation of Gynecology and Obstetrics
GnRH: Gonadotropin releasing hormone
HMGA2: High mobility group AT-hook 2
HPF: High-power field
IAEL: Intra-abdominal extrauterine leiomyoma
IVC: Inferior vena cava
IVL: Intravascular leiomyoma
LMS: Leiomyosarcoma
MRI: Magnetic resonance imaging
PEComa: Perivascular epithelioid cell tumor
US: Ultrasound
