# Supplementary material for: Diagnosis, treatment and clinical outcomes of extrauterine sites of leiomyomatosis: a systematic review
Source: Ann Med. 2025 Aug 21;57(1):2546681. doi: 10.1080/07853890.2025.2546681 (PMC12372508; doi:10.1080/07853890.2025.2546681)
Supplement: Supplementary File S2 ESL ANNALS.docx [file IANN_A_2546681_SM9293.docx]

**Supplementary File 2 – S2**

**“Diagnosis, treatment and clinical outcomes of extrauterine sites of leiomyomatosis: a systematic review.”**

| **Supplementary Table S8** | Patient’s BMI, race, type of previous surgery | pag. 2 |
| --- | --- | --- |
| **Supplementary Table S9** | Immunohistochemistry after surgery performed before diagnosis of ESLs | pag. 3 |
| **Supplementary Table S10** | Immunohistochemistry after surgery for ESLs | pag. 4 |
| **Supplementary Table S11** | Comparison of patient characteristics between IAELs and EAELs cases | pag. 5 |
| **Supplementary Table S12** | Comparison of ESLs characteristics between IAELs and EAELs cases | pag. 6 |
| **Supplementary Table S13** | Comparison of management strategies and outcomes between IAELs and EAELs cases | pag. 7 |
| **Supplementary Figure S1** | Quality assessment of included case reports | pag. 8 |
| **Supplementary Figure S2** | Quality assessment of included case series | pag. 9 |

| **Supplementary Table S8.** Patient’s BMI, race, type of previous surgery. | | | | | | |  |
| --- | --- | --- | --- | --- | --- | --- | --- |
|  | **Intra-abdominal lesions** | | | **Extra-abdominal lesions** | | | |
|  | **DPL^1^** | **PM^2^** | **Total** | **BML^3^** | **IVL^4^** | **Total** | |
| **Characteristics** | **93 cases** | **30 cases** | **123 cases** | **155 cases** | **94 cases** | **249 cases** | |
| **BMI^5^** |  |  |  |  |  |  | |
| < 30 kg/m^2^ | 5/7 (71.4%) | 0/3 (-) | 5/10 (50%) | 2/11 (18.2%) | 3/9 (33.3%) | 5/10 (50%) | |
| 30-34,9 kg/m^2^ | 0/7 (-) | 1/3 (33.3%) | 1/10 (10%) | 5/11 (45.4%) | 4/9 (44.5%) | 9/10 (90%) | |
| 35-39,9 kg/m^2^ | 1/7 (14.3%) | 0/3 (-) | 1/2 (50%) | 1/11 (9.1%) | 0/9 (-) | 1/2 (50%) | |
| ≥40 kg/m^2^ | 1/7 (14.3%) | 2/3 (66.7%) | 3/8 (37.5%) | 3/11 (27.3%) | 2/9 (22.2%) | 5/8 (62.5%) | |
| **Race** |  |  |  |  |  |  | |
| American Indian or Alaska native | 0/22 (-) | 0/12 (-) | 0/0 (-) | 0/44 (-) | 0/24 (-) | 0/0 (-) | |
| Asian | 10/22 (45.5%) | 5/12 (41.7%) | 15/42 (35.7%) | 18/44 (40.9%) | 9/24 (37.5%) | 27/42 (64.3%) | |
| Black or African American | 1/22 (4.5%) | 1/12 (8.3%) | 2/13 (15.4%) | 7/44 (15.9%) | 4/24 (16.7%) | 11/13 (85.6%) | |
| Hispanic or Latino | 0/22 (-) | 1/12 (8.3%) | 1/7 (14.3%) | 5/44 (11.4%) | 1/24 (4.1%) | 6/7 (85.7%) | |
| Native Hawaiian or other pacific islander | 0/22 (-) | 0/12 (-) | 0/1 (-) | 1/44 (2.3%) | 0/24 (-) | 1/1 (100%) | |
| White | 11/22 (50%) | 5/12 (41.7%) | 16/39 (41.1%) | 13/44 (29.5%) | 10/24 (41.7%) | 23/39 (58.9%) | |
| **Previous surgery**^*^ |  |  |  |  |  |  | |
| None | 25/93 (26.9%) | 7/30 (23.3%) | 32/84 (38,1%) | 15/155 (9.7%) | 37/94 (39.4%) | 52/84 (61,9%) | |
| Myomectomy | 31/93 (33.3%) | 5/30 (16.7%) | 36/93 (38,71%) | 43/155 (27.8%) | 14/94 (14.9%) | 57/93 (61,29%) | |
| *Laparoscopic* | 19/31 (61.3%) | 0/5 (-) | 19/22 (86,36%) | 2/43 (4.7%) | 1/14 (7.1%) | 3/22 (13,64%) | |
| *Laparotomic* | 6/31 (19.3%) | 4/5 (80.0%) | 10/21 (47,62%) | 9/43 (20.9%) | 2/14 (14.3%) | 11/21 (52,38%) | |
| *Hysteroscopic* | 2/31 (6.5%) | 0/5 (-) | 2/4 (50%) | 1/43 (2.3%) | 1/14 (7.1%) | 2/4 (50%) | |
| *Unknown* | 4/31 (12.9%) | 1/5 (20.0%) | 5/37 (13.5%) | 31/43 (72.1%) | 10/14 (71.5%) | 32/37 (86.5%) | |
| Hysterectomy | 28/93 (30,1%) | 8/30 (26.7%) | 36/150 (24.0%) | 82/155 (52.9%) | 32/94 (34.0%) | 114/150 (76.0%) | |
| *Laparoscopic* | 6/28 (21.4%) | 3/8 (37.5%) | 9/15 (60.0%) | 4/82 (4,9%) | 2/32 (6.3%) | 6/15 (40.0%) | |
| *Laparotomic* | 13/28 (46.4%) | 4/8 (50.0%) | 17/58 (29.3%) | 28/82 (34,0%) | 13/32 (40.6%) | 41/58 (70.7%) | |
| *Unknown* | 9/28 (32.2%) | 1/8 (12.5%) | 10/77 (12.9%) | 50/82 (60,1%) | 17/32 (53.1%) | 67/77 (87.1%) | |
| SH^6^ | 1/93 (1,1%) | 1/30 (3.3%) | 2/13 (15.4%) | 9/155 (5.8%) | 2/94 (2.1%) | 11/13 (84.6%) | |
| *Laparoscopic* | 1/1 (100.0%) | 1/1 (100.0%) | 2/2 (100%) | 0/9 (-) | 0/2 (-) | 0/2 (-) | |
| *Laparotomic* | 0/1 (-) | 0/1 (-) | 0/2 (-) | 2/9 (22.2%) | 0/2 (-) | 2/2 (100%) | |
| *Unknown* | 0/1 (-) | 0/1 (-) | 0/9 (-) | 7/9 (77.8%) | 2/2 (100.0%) | 9/9 (100%) | |
| Cesarean section | 4/93 (4.3%) | 4/30 (13.3%) | 8/18 (44.5%) | 5/155 (3.2%) | 5/94 (5.3%) | 8/18 (44.5%) | |
| Non-gynaecological surgery | 4/93 (4.3%) | 5/30 (16.7%) | 9/14 (64.3%) | 1/155 (0.6%) | 4/94 (4.3%) | 5/14 (35.7%) | |
| *Laparoscopic* | 0/4 (-) | 2/5 (40.0%) | 2/3 (66.7%) | 0/1 (-) | 1/4 (25.0%) | 1/3 (33.3%) | |
| *Laparotomic* | 2/4 (50.0%) | 1/5 (20.0%) | 3/3 (100%) | 0/1 (-) | 0/4 (-) | 0/3 (-) | |
| *Others* | 1/4 (25.0%) | 1/5 (20.0%) | 2/3 (66.7%) | 0/1 (-) | 1/4 (25.0%) | 1/3 (33.3%) | |
| *Unknown* | 1/4 (25.0%) | 1/5 (20.0%) | 2/5 (40.0%) | 1/1 (100.0%) | 2/4 (50.0%) | 3/5 (60.0%) | |
| ^*^Previous surgery before diagnosis of ESLs; ^1^DPL: disseminated peritoneal leiomyomatosis; ^2^PM: parasitic myoma; ^3^BML: benign metastasizing leiomyomatosis; ^4^IVL: intravascular leiomyomatosis; ^5^BMI: body mass index; ^6^SH: supracervical hysterectomy. | | | | | | |  |

| **Supplementary Table S9.** Immunohistochemistry after surgery performed before diagnosis of ESLs. | | | | | | |  |
| --- | --- | --- | --- | --- | --- | --- | --- |
|  | **Intra-abdominal lesions** | | | **Extra-abdominal lesions** | | | |
|  | **DPL^1^** | **PM^2^** | **Total** | **BML^3^** | **IVL^4^** | **Total** | |
| **Characteristics** | **93 cases** | **30 cases** | **123 cases** | **155 cases** | **94 cases** | **249 cases** | |
| **Desmina** |  |  |  |  |  |  | |
| Yes | - | - | 0/4 (-) | 3/140 (100%) | 1/57 (100%) | 4/4 (100%) | |
| No | - | - | - | - | - | - | |
| **H-caldesmone** |  |  |  |  |  |  | |
| Yes | - | - | - | - | - | - | |
| No | - | - | - | - | - | - | |
| **SMA^5^** |  |  |  |  |  |  | |
| Yes | - | 1/23 (100%) | 1/4 (25%) | 3/140 (100%) | - | 3/4 (75%) | |
| No | - | - | - | - | - | - | |
| **MSA^6^** |  |  |  |  |  |  | |
| Yes | - | - | - | - | - | - | |
| No | - | - | - | - | - | - | |
| **Calponin** |  |  |  |  |  |  | |
| Yes | - | - | 0/1 (-) | 1/140 (100%) | - | 1/1 (100%) | |
| No | - | - | - | - | - | - | |
| **SMMHC^7^** |  |  |  |  |  |  | |
| Yes | - | - | - | - | - | - | |
| No | - | - | - | - | - | - | |
| **ER^8^** |  |  |  |  |  |  | |
| Yes | - | 1/23 (100%) | 1/5 (20%) | 4/140 (100%) | - | 4/5 (80%) | |
| No | - | - | - | - | - | - | |
| **PR^9^** |  |  |  |  |  |  | |
| Yes | - | 1/23 (100%) | 1/4 (25%) | 3/140 (100%) | - | 3/4 (75%) | |
| No | - | - | - | - | - | - | |
| **WT1^10^** |  |  |  |  |  |  | |
| Yes | - | - | - | - | - | - | |
| No | - | - | - | - | - | - | |
| **Oxytocin receptor** |  |  |  |  |  |  | |
| Yes | - | - | - | - | - | - | |
| No | - | - | - | - | - | - | |
| ^1^DPL: disseminated peritoneal leiomyomatosis; ^2^PM: parasitic myoma; ^3^BML: benign metastasizing leiomyomatosis; ^4^IVL: intravascular leiomyomatosis; ^5^SMA: smooth muscle actin; ^6^MSA: muscle specific actin; ^7^SMMHC: smooth muscle myosin heavy chain; ^8^ER: estrogen receptor; ^9^PR: progesterone receptor; ^10^WT1: Wilms tumor 1. | | | | | | |  |

| **Supplementary Table S10.** Immunohistochemistry after surgery for ESLs. | | | | | | |  |
| --- | --- | --- | --- | --- | --- | --- | --- |
|  | **Intra-abdominal lesions** | | | **Extra-abdominal lesions** | | | |
|  | **DPL^1^** | **PM^2^** | **Total** | **BML^3^** | **IVL^4^** | **Total** | |
| **Characteristics** | **93 cases** | **30 cases** | **123 cases** | **155 cases** | **94 cases** | **249 cases** | |
| **Desmina** |  |  |  |  |  |  | |
| Yes | 34/36 (94.4%) | 3/3 (100.0%) | 37/146 (25.3%) | 76/76 (100.0%) | 33/34 (97.1%) | 109/146 (74.7%) | |
| No | 2/36 (5.6%) | 0/3 (-) | 2/3 (66.7%) | 0/76 (-) | 1/34 (2.9%) | 1/3 (33.3%) | |
| **H-caldesmone** |  |  |  |  |  |  | |
| Yes | 10/10 (100.0%) | 2/2 (100.0%) | 12/39 (30.7%) | 16/16 (100.0%) | 11/13 (84.6%) | 27/39 (69.3%) | |
| No | 0/10 (-) | 0/2 (-) | 0/2 (-) | 0/16 (-) | 2/13 (15.4%) | 2/2 (100%) | |
| **SMA^5^** |  |  |  |  |  |  | |
| Yes | 42/43 (97.7%) | 9/9 (100.0%) | 51/180 (28.3%) | 89/89 (100.0%) | 40/40 (100.0%) | 129/180 (71.7%) | |
| No | 1/43 (2.3%) | 0/9 (-) | 1/1 (100%) | 0/89 (-) | 0/40 (-) | 0/1 (-) | |
| **MSA^6^** |  |  |  |  |  |  | |
| Yes | 1/1 (100.0%) | - | 1/8 (12.5%) | 5/7 (71.4%) | 2/2 (100.0%) | 7/8 (87.5%) | |
| No | 0/1 (-) | - | 0/2 (-) | 2/7 (28.6%) | 0/2 (-) | 2/2 (100%) | |
| **Calponin** |  |  |  |  |  |  | |
| Yes | - | - | 0/3 (-) | 3/3 (100.0%) | - | 3/3 (100%) | |
| No | - | - | - | - | - | - | |
| **SMMHC^7^** |  |  |  |  |  |  | |
| Yes | - | - | - | - | - | - | |
| No | - | - | - | - | - | - | |
| **ER^8^** |  |  |  |  |  |  | |
| Yes | 34/39 (87.2%) | 5/5 (100.0%) | 39/154 (25.3%) | 89/95 (93.7%) | 26/27 (96.3%) | 115/154 (74.7%) | |
| No | 5/39 (12.8%) | 0/5 (-) | 5/12 (41.7%) | 6/95 (6.3%) | 1/27 (3.7%) | 7/12 (58.3%) | |
| **PR^9^** |  |  |  |  |  |  | |
| Yes | 33/38 (86.8%) | 3/3 (100.0%) | 36/149 (24.2%) | 89/90 (98.9%) | 24/24 (100.0%) | 113/149 (75.8%) | |
| No | 5/38 (13.2%) | 0/3 (-) | 5/6 (83.3%) | 1/89 (1.1%) | 0/24 (-) | 1/6 (16.7%) | |
| **WT1^10^** |  |  |  |  |  |  | |
| Yes | - | - | - | - | - | - | |
| No | - | - | - | - | - | - | |
| **Oxytocin receptor** |  |  |  |  |  |  | |
| Yes | - | - | - | - | - | - | |
| No | - | - | - | - | - | - | |
| ^1^DPL: disseminated peritoneal leiomyomatosis; ^2^PM: parasitic myoma; ^3^BML: benign metastasizing leiomyomatosis; ^4^IVL: intravascular leiomyomatosis; ^5^SMA: smooth muscle actin; ^6^MSA: muscle specific actin; ^7^SMMHC: smooth muscle myosin heavy chain; ^8^ER: estrogen receptor; ^9^PR: progesterone receptor; ^10^WT1: Wilms tumor 1. | | | | | | |  |

| **Supplementary Table S11.** Comparison of patient characteristics between IAELs^1^ and EAELs^2^ cases. | | | |
| --- | --- | --- | --- |
|  | **Total^§^** | **IAEL** | **EAEL** |
|  | **272 cases** | **123 cases** | **249 cases** |
| **Characteristics** | **n** | **n (%)** | **n (%)** |
| **Parity** |  |  |  |
| Nulliparous | 53 | 33 (61.3) | 20 (39.7) |
| Multiparous | 123 | 51(41.5) | 72 (58.5) |
| **Symptom (predominant)** |  |  |  |
| Dysmenorrhea | 2 | 2 (100) | 0 (-) |
| Abdominal/pelvic pain | 118 | 73 (61.8) | 45 (38.2) |
| Compression of adjacent organs | 19 | 8 (42.1) | 11 (57.9) |
| Asthenia | 6 | 1 (16.6) | 5 (83.4) |
| Dyspnea | 68 | 1 (1.5) | 67 (98.5) |
| Thrombosis-related | 17 | 0 (-) | 17 (100) |
| Bone-related | 8 | 1 (12.5) | 7 (87.5) |
| No symptoms | 99 | 26 (26.3) | 73 (73.7) |
| **Sign (predominant)** |  |  |  |
| Pelvic mass | 72 | 40 (55.5) | 32 (45.5) |
| Vaginal bleeding | 35 | 13 (37.1) | 22 (62.9) |
| Abdominal distension | 39 | 23 (58.9) | 16 (41.1) |
| No signs | 136 | 24 (17.6) | 112 (82.4) |
| **Previous surgery**^†^ |  |  |  |
| None | 84 | 32 (38,1) | 52 (61,9) |
| Myomectomy | 93 | 36 (38,71) | 57 (61,29) |
| Hysterectomy | 150 | 36 (24.0) | 114 (76.0) |
| SH^5^ | 13 | 2 (15.4) | 11 (84.6) |
| Cesarean section | 18 | 8 (44.5) | 8 (44.5) |
| Non-gynaecological surgery | 14 | 9 (64.3) | 5 (35.7) |
| **Morcellation** |  |  |  |
| Yes | 17 | 16 (94.1) | 1 (5.9) |
| No or in a bag | 51 | 5 (9.8) | 46 (90.2) |
| Only cases with available data are reported. ^§^Total: IAEL cases + EAEL cases. ^1^IAEL: intra-abdominal extrauterine leiomyoma; ^2^EAEL: extra-abdominal extrauterine leiomyoma; ^3^MT: medical treatment; ^4^GnRH: gonadotropin releasing hormone. | | | |

| **Supplementary Table S12.** Comparison of ESLs^1^ characteristics between IAELs^2^ and EAELs^3^ cases. | | | |
| --- | --- | --- | --- |
|  | **Total^§^** | **IAEL** | **EAEL** |
|  | **272 cases** | **123 cases** | **249 cases** |
| **Characteristics** | **n** | **n (%)** | **n (%)** |
| **Diagnostic tools**^†^ |  |  |  |
| US^4^ | 95 | 41 (43.2) | 54 (56.8) |
| MR^5^ | 40 | 17 (42.5) | 23 (75.5) |
| CT^6^ | 153 | 38 (24.8) | 115 (75.2) |
| Surgery | 25 | 22 (88.0) | 3 (12.0) |
| Others | 49 | 1 (2.1) | 48 (97.9) |
| **Mitosis** |  |  |  |
| 0-4 | 272 | 87 (31.9) | 185 (68.1) |
| 5-9 | 19 | 8 (42.1) | 11 (57.9) |
| ≥10 | 7 | 5 (71.4) | 2 (28.6) |
| **Atypia** |  |  |  |
| None | 229 | 75 (32.7) | 154 (67.3) |
| Mild | 42 | 15 (35.7) | 27 (64.3) |
| Moderate | 3 | 1 (33.3) | 2 (66.7) |
| Severe | 5 | 3 (60.0) | 2 (40.0) |
| **Necrosis** |  |  |  |
| Absent | 190 | 60 (31.6) | 130 (68.4) |
| Present | 31 | 14 (45.2) | 17 (54.8) |
| Only cases with available data are reported. ^§^Total: IAEL cases + EAEL cases. ^1^ESL: extrauterine sites of leiomyomatosis; ^2^IAEL: intra-abdominal extrauterine leiomyoma; ^3^EAEL: extra-abdominal extrauterine leiomyoma; ^4^US: Ultrasound; ^5^MR: magnetic resonance; ^6^CT: computed tomography. | | | |

| **Supplementary Table S13.** Comparison between IAELs^1^ and EAELs^2^ cases: management strategies and outcomes. | | | |
| --- | --- | --- | --- |
|  | **Total^§^** | **IAEL** | **EAEL** |
|  | **272 cases** | **123 cases** | **249 cases** |
| **Characteristics** | **n** | **n (%)** | **n (%)** |
| **Management** |  |  |  |
| Surgery alone | 254 | 97 (38.2) | 157 (61.8) |
| MT^3^ alone | 14 | 5 (35.7) | 9 (64.3) |
| Surgery + MT | 95 | 21 (22.1) | 74 (77.9) |
| Follow-up alone | 9 | 0 (-) | 9 (100) |
| **Surgical access** |  |  |  |
| Laparoscopic | 25 | 20 (80.0) | 5 (20.0) |
| Laparotomic | 173 | 83 (47.9) | 90 (52.1) |
| Hysteroscopic |  | - | - |
| Others | 119 | 8 (6.7) | 111 (93.3) |
| **Medical treatment** |  |  |  |
| No | 263 | 97 (36.9) | 166 (63.1) |
| Mifepristone |  | - | - |
| Ulipristal acetate | 3 | 1 (25.0) | 3 (75.0) |
| Analogues GnRH^4^ | 52 | 11 (21.1) | 41 (78.9) |
| Others | 53 | 14 (26.4) | 39 (73.6) |
| **Outcomes** |  |  |  |
| Death | 11 | 5 (45.5) | 6/ (54.5) |
| Less symptoms | 17 | 5 (29.4) | 12 (70.6) |
| No symptoms | 178 | 71 (39.9) | 107 (60.1) |
| Size mass decreased | 32 | 11 (34.4) | 21 (65.6) |
| No symptoms and size mass decreased | 53 | 8 (15.1) | 45 (84.9) |
| Size mass decreased and less symptoms | 15 | 2 (13.3) | 13 (86.7) |
| Only cases with available data are reported. ^§^Total: IAEL cases + EAEL cases. ^1^IAEL: intra-abdominal extrauterine leiomyoma; ^2^EAEL: extra-abdominal extrauterine leiomyoma; ^3^MT: medical treatment; ^4^GnRH: gonadotropin releasing hormone. | | | |

**Supplementary Figure S1.** Quality assessment of included case reports based on JBI (Joanna Briggs Institute) critical appraisal checklist for case reports.

**
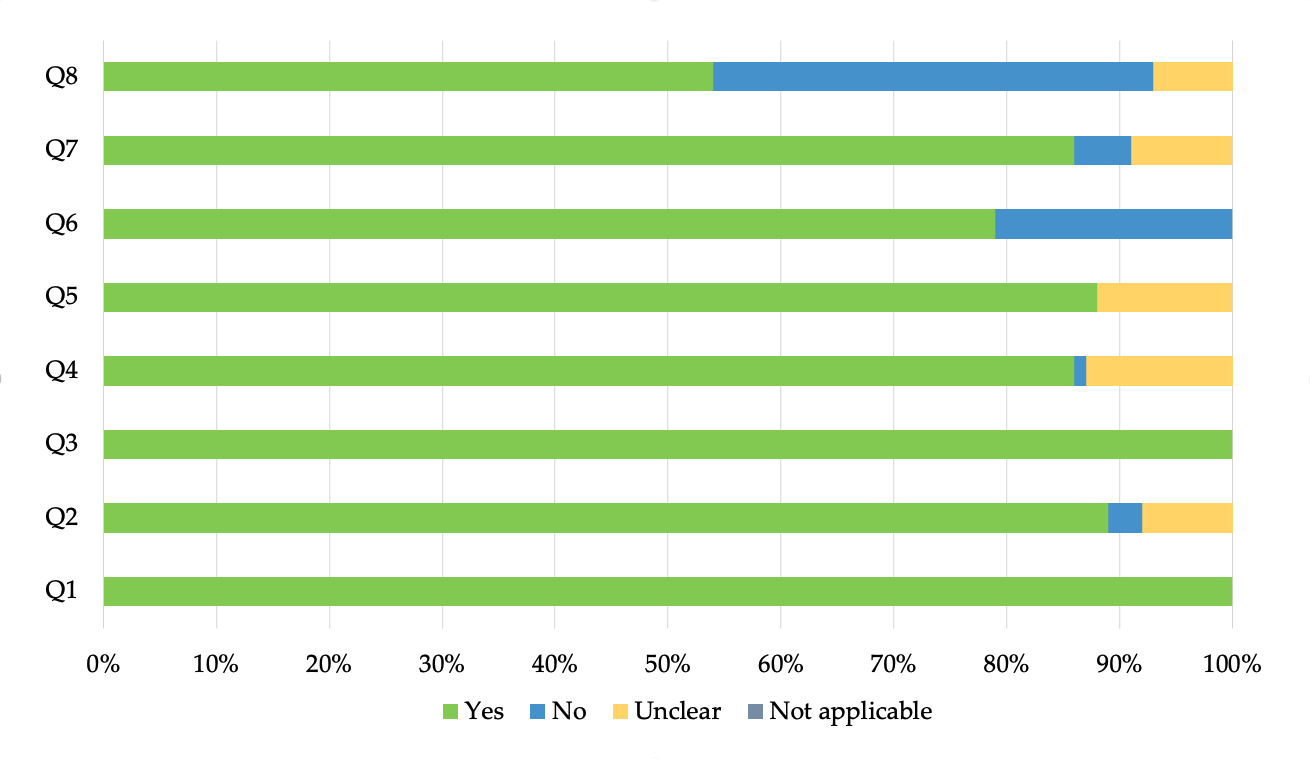
**

|  | **Q1** | **Q2** | **Q3** | **Q4** | **Q5** | **Q6** | **Q7** | **Q8** |
| --- | --- | --- | --- | --- | --- | --- | --- | --- |
| **Yes** | 345 | 306 | 344 | 295 | 304 | 273 | 295 | 186 |
| **No** | 0 | 9 | 1 | 3 | 0 | 72 | 18 | 133 |
| **Unclear** | 0 | 30 | 0 | 47 | 41 | 0 | 32 | 26 |
| **Not Applicable** | 0 | 0 | 0 | 0 | 0 | 0 | 0 | 0 |
| **Q1.** Were patient’s demographic characteristics clearly described?  **Q2.** Was the patient’s history clearly described and presented as a timeline?  **Q3.** Was the current clinical condition of the patient on presentation clearly described?  **Q4.** Were diagnostic tests or assessment methods and the results clearly described?  **Q5.** Was the intervention(s) or treatment procedure(s) clearly described?  **Q6.** Was the post-intervention clinical condition clearly described?  **Q7.** Were adverse events (harms) or unanticipated events identified and described?  **Q8.** Does the case report provide takeaway lessons? | | | | | | | | |

**
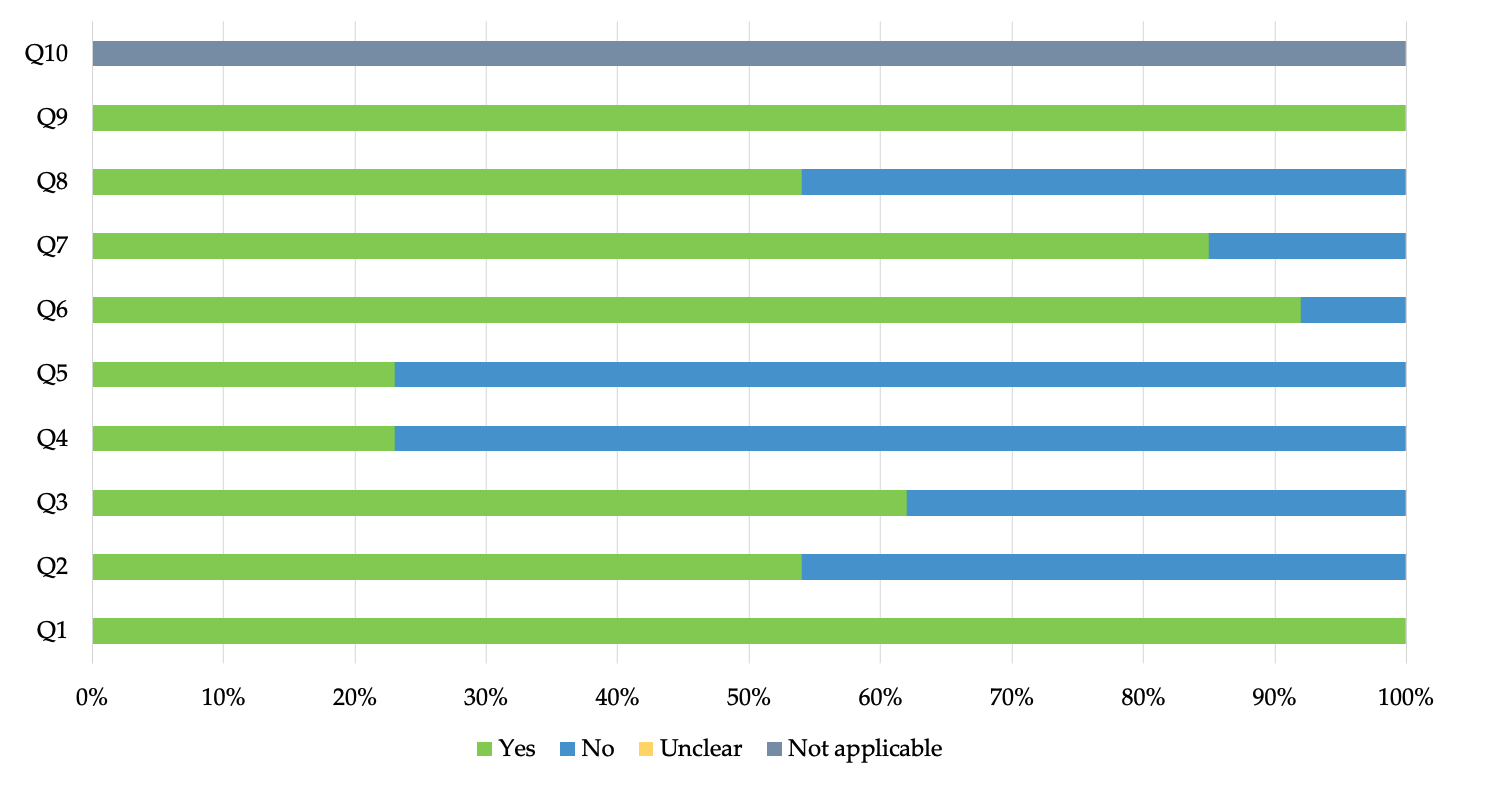
Supplementary Figure S2.** Quality assessment of included case series based on JBI (Joanna Briggs Institute) critical appraisal checklist for case series.

|  | **Q1** | **Q2** | **Q3** | **Q4** | **Q5** | **Q6** | **Q7** | **Q8** | **Q9** | **Q10** |
| --- | --- | --- | --- | --- | --- | --- | --- | --- | --- | --- |
| **Yes** | 13 | 7 | 8 | 3 | 3 | 12 | 11 | 7 | 13 | 0 |
| **No** | 0 | 6 | 5 | 10 | 10 | 1 | 2 | 6 | 0 | 0 |
| **Unclear** | 0 | 0 | 0 | 0 | 0 | 0 | 0 | 0 | 0 | 0 |
| **Not Applicable** | 0 | 0 | 0 | 0 | 0 | 0 | 0 | 0 | 0 | 13 |
| **Q1.** Were there clear criteria for inclusion in the case series?  **Q2.** Was the condition measured in a standard, reliable way for all participants included in the case series?  **Q3.** Were valid methods used for identification of the condition for all participants included in the case series?  **Q4.** Did the case series have consecutive inclusion of participants?  **Q5.** Did the case series have complete inclusion of participants?  **Q6.** Was there clear reporting of the demographics of the participants in the study?  **Q7.** Was there clear reporting of clinical information of the participants?  **Q8.** Were the outcomes or follow up results of cases clearly reported?  **Q9.** Was there clear reporting of the presenting site(s)/clinic(s) demographic information?  **Q10.** Was statistical analysis appropriate? | | | | | | | | | | |
