# Supplementary material for: Diagnosis, treatment and clinical outcomes of extrauterine sites of leiomyomatosis: a systematic review
Source: Ann Med. 2025 Aug 21;57(1):2546681. doi: 10.1080/07853890.2025.2546681 (PMC12372508; doi:10.1080/07853890.2025.2546681)
Supplement: Supplementary File S1 ESL ANNALS.docx [file IANN_A_2546681_SM9292.docx]

**Supplementary File 1 – S1**

**“Diagnosis, treatment and clinical outcomes of extrauterine sites of leiomyomatosis: a systematic review.”**

| **Supplementary Table S1** | Pubmed search strategy | pag. 2 |
| --- | --- | --- |
| **Supplementary Table S2** | Scopus search strategy | pag. 5 |
| **Supplementary Table S3** | Web of Science search strategy | pag. 7 |
| **Supplementary Table S4** | Disseminated peritoneal leiomyomatosis (DPL) data | pag. 10 |
| **Supplementary Table S5** | Parasitic myoma (PM) data | pag. 18 |
| **Supplementary Table S6** | Benign metastastic leiomyoma (BML) data | pag. 21 |
| **Supplementary Table S7** | Intravascular leiomyoma (IVL) data | pag. 36 |

| **Supplementary Table S1: Pubmed search strategy** | | | | | |
| --- | --- | --- | --- | --- | --- |
| **Search date:** | | **17/01/2024** | |  |  |
| **#Number** | **Term #1** | | **Full search line (synonims, plurals, different forms, MH)** | **Revised search line** | **# Reports** |
| **#1** | Benign [ALL] | | "benign"[ALL] OR "benignancies"[ALL] OR "benignancy"[ALL] OR "benignant"[ALL] OR "benigne"[ALL] OR "benignity"[ALL] OR "benigns"[ALL] | “benign*”[TW] |  |
| **#2** | Benign [MH] | | - | - |  |
| **#3** | Metastasizing [ALL] | | "metastasation"[ALL] OR "metastasic"[ALL] OR "metastasing"[ALL] OR "metastasise"[ALL] OR "metastasised"[ALL] OR "metastasises"[ALL] OR "metastasising"[ALL] OR "metastasization"[ALL] OR "metastasizes"[ALL] OR "metastasizing"[ALL] OR ("neoplasm"[ALL] AND "metastasis"[ALL]) OR "neoplasm metastasis"[ALL] OR "metastase"[ALL] OR "metastases"[ALL] OR "metastasize"[ALL] OR "metastasized"[ALL] | “metastas*”[TW] |  |
| **#4** | Metastasis [MH] | | "neoplasm metastasis"[MH] OR “lymphatic metastasis”[MH] | "neoplasm metastasis"[MH] OR “lymphatic metastasis”[MH] |  |
| **#5** | Leiomyoma [ALL] OR myoma [ALL] OR leiomyomatosis [ALL] OR fibroid [ALL] | | "leiomyoma"[ALL] OR "leiomyomas"[ALL] OR "myoma"[ALL] OR "myomas"[ALL] OR "myoma’s"[ALL] OR "leiomyomatosis"[ALL] OR "leiomyomatoses"[ALL] OR "leiomyoma"[ALL] OR "fibroid’s"[ALL] OR "fibroid"[ALL] OR "fibroids"[ALL] | “leiomyoma*”[TW] OR “myoma*”[TW] OR “fibroid*”[TW] OR “fibroid tumor*”[TW] |  |
| **#6** |  | | "leiomyoma"[MH] OR "myoma"[MH] OR "leiomyomatosis"[MH] | "leiomyoma"[MH] OR "myoma"[MH] OR "leiomyomatosis"[MH] |  |
| **#7** | (#1 OR #2) AND (#3 OR #4) AND (#5 OR #6) | | "benign*"[TW] AND ("metastas*"[TW] OR ("neoplasm metastasis"[MH] OR "lymphatic metastasis"[MH])) AND ("leiomyoma*"[TW] OR "myoma*"[TW] OR "fibroid*"[TW] OR "fibroid tumor*"[TW] OR ("leiomyoma"[MH] OR "myoma"[MH] OR "leiomyomatosis"[MH])) |  |  |
| **#8** | Intravascular [ALL] | | "intravascular"[ALL] | “intravascular*”[TW] |  |
| **#9** | Intravascular [MH] | | - | - |  |
| **#10** | Intravenous [ALL] | | "intraveneous"[ALL] OR "intraveneously"[ALL] OR "intravenous"[ALL] OR "intravenously"[ALL] | “intraven*”[TW] |  |
| **#11** | Intravenous [MH] | | - | - |  |
| **#12** | Leiomyoma [ALL] OR myoma [ALL] OR leiomyomatosis [ALL] OR fibroid [ALL] | | #5 OR #6 | ("leiomyoma*"[TW] OR "myoma*"[TW] OR "fibroid*"[TW] OR "fibroid tumor*"[TW] OR ("leiomyoma"[MH] OR "myoma"[MH] OR "leiomyomatosis"[MH])) |  |
| **#13** | (#8 OR #9) OR (#10 OR #11) AND #12 | | “intravascular*”[TW] OR “intraven*”[TW] AND ("leiomyoma*"[TW] OR "myoma*"[TW] OR "fibroid*"[TW] OR "fibroid tumor*"[TW] OR ("leiomyoma"[MH] OR "myoma"[MH] OR "leiomyomatosis"[MH])) |  |  |
| **#14** | Disseminated [ALL] | | "disseminate"[ALL] OR "disseminated"[ALL] OR "disseminates"[ALL] OR "disseminating"[ALL] OR "dissemination"[ALL] OR "disseminations"[ALL] OR "disseminator"[ALL] OR "disseminators"[ALL] | “disseminat*”[TW] |  |
| **#15** | Disseminated [MH] OR Dissemination [MH] | | - | - |  |
| **#16** | Diffused [ALL] | | "diffusable"[ALL] OR "diffusant"[ALL] OR "diffusants"[ALL] OR "diffuse"[ALL] OR "diffusely"[ALL] OR "diffuses"[ALL] OR "diffusibility"[ALL] OR "diffusible"[ALL] OR "diffusion"[ALL] OR "diffused"[ALL] OR "diffusing"[ALL] OR "diffusions"[ALL] OR "diffusive"[ALL] OR "diffusively"[ALL] OR "diffusivities"[ALL] OR "diffusivity"[ALL] | “diffuse*”[TW] |  |
| **#17** | Diffused [MH] OR Diffusion [MH] | | ~~"diffusion"[MH]~~ (not usable due to different topic) | - |  |
| **#18** | Peritoneal[ALL] | | “peritoneally"[ALL] OR "peritoneum"[ALL] OR "peritoneal"[ALL] OR "peritonism"[ALL] OR "peritonitis"[ALL] | “periton*”[TW] |  |
| **#19** | Peritoneal[MH] | | "peritoneum"[MAJR] | "peritoneum"[MAJR] |  |
| **#20** | Leiomyoma [ALL] OR myoma [ALL] OR leiomyomatosis [ALL] OR fibroid [ALL] | | #12 | ("leiomyoma*"[TW] OR "myoma*"[TW] OR "fibroid*"[TW] OR "fibroid tumor*"[TW] OR ("leiomyoma"[MH] OR "myoma"[MH] OR "leiomyomatosis"[MH])) |  |
| **#21** | (#14 OR #15) OR (#16 OR #17) AND (#18 OR #19) AND #12 | | “disseminat*”[TW] OR “diffuse*”[TW] AND (“periton*”[TW] OR "peritoneum"[MAJR]) AND ("leiomyoma*"[TW] OR "myoma*"[TW] OR "fibroid*"[TW] OR "fibroid tumor*"[TW] OR ("leiomyoma"[MH] OR "myoma"[MH] OR "leiomyomatosis"[MH])) |  |  |
| **#22** | Parasitic [ALL] | | "parasitation"[ALL] OR "parasited"[ALL] OR "parasites"[MH] OR "parasites"[ALL] OR "parasite"[ALL] OR "parasitical"[ALL] OR "parasiticALLy"[ALL] OR "parasitics"[ALL] OR "parasiting"[ALL] OR "parasitism"[ALL] OR "parasitisms"[ALL] OR "parasitization"[ALL] OR "parasitize"[ALL] OR "parasitized"[ALL] OR "parasitizes"[ALL] OR "parasitizing"[ALL] OR "parasitic"[ALL] | “parasit*”[TW] |  |
| **#23** | Parasitic [MH] | | ~~"parasites"[MH] OR "parasitology"[MH] OR "parasitology"[MeSH Subheading]~~ (not usable due to different topic) | - |  |
| **#24** | Leiomyoma [ALL] OR myoma [ALL] OR leiomyomatosis [ALL] OR fibroid [ALL] | | #12 | ("leiomyoma*"[TW] OR "myoma*"[TW] OR "fibroid*"[TW] OR "fibroid tumor*"[TW] OR ("leiomyoma"[MH] OR "myoma"[MH] OR "leiomyomatosis"[MH])) |  |
| **#25** | (#22 OR #23) AND #12 | | “parasit*”[TW] AND ("leiomyoma*"[TW] OR "myoma*"[TW] OR "fibroid*"[TW] OR "fibroid tumor*"[TW] OR ("leiomyoma"[MH] OR "myoma"[MH] OR "leiomyomatosis"[MH])) |  |  |
| **#26** | #7 OR #13 OR #21 OR #25 | | ("benign*"[TW] AND ("metastas*"[TW] OR ("neoplasm metastasis"[MH] OR "lymphatic metastasis"[MH]))) OR (“intravascular*”[TW] OR “intraven*”[TW]) OR (“disseminat*”[TW] OR “diffuse*”[TW] AND (“periton*”[TW] OR "peritoneum"[MAJR])) OR (“parasit*”[TW]) AND ("leiomyoma*"[TW] OR "myoma*"[TW] OR "fibroid*"[TW] OR "fibroid tumor*"[TW] OR ("leiomyoma"[MH] OR "myoma"[MH] OR "leiomyomatosis"[MH])) |  |  |
| **#27** | English language | | (("benign*"[TW] AND ("metastas*"[TW] OR ("neoplasm metastasis"[MH] OR "lymphatic metastasis"[MH]))) OR ("intravascular*"[TW] OR "intraven*"[TW]) OR (("disseminat*"[TW] OR "diffuse*"[TW]) AND ("periton*"[TW] OR "peritoneum"[MeSH Major Topic])) OR "parasit*"[TW]) AND ("leiomyoma*"[TW] OR "myoma*"[TW] OR "fibroid*"[TW] OR "fibroid tumor*"[TW] OR ("leiomyoma"[MH] OR "myoma"[MH] OR "leiomyomatosis"[MH])) AND "english"[Language] |  |  |

| **Supplementary Table S2: Scopus search strategy** | | | | | |
| --- | --- | --- | --- | --- | --- |
| **Search date:** | | **17/01/2024** | |  |  |
| **#Number** | **Term #1** | | **Pubmed search line** | **Translated search line to Scopus** | **# Reports** |
| **#1** | Benign [ALL] | | “benign*”[TW] | TITLE-ABS-KEY(benign*) |  |
|  | Benign [MH] | | - | - |  |
| **#2** | Metastasizing [ALL] | | “metastas*”[TW] | TITLE-ABS-KEY(metastas*) |  |
|  | Metastasis [MH] | | "neoplasm metastasis"[MH] OR “lymphatic metastasis”[MH] | - |  |
| **#3** | Leiomyoma [ALL] OR myoma [ALL] OR leiomyomatosis [ALL] OR fibroid [ALL] | | “leiomyoma*”[TW] OR “myoma*”[TW] OR “fibroid*”[TW] OR “fibroid tumor*”[TW] | TITLE-ABS-KEY(leiomyoma*) OR TITLE-ABS-KEY(myoma*) OR TITLE-ABS-KEY(fibroid*) OR TITLE-ABS-KEY("fibroid tumor*") |  |
|  |  | | "leiomyoma"[MH] OR "myoma"[MH] OR "leiomyomatosis"[MH] | - |  |
| **#4** | #1 AND #2 AND #3 | |  | ( TITLE-ABS-KEY ( benign* ) ) AND ( TITLE-ABS-KEY ( metastas* ) ) AND ( TITLE-ABS-KEY ( leiomyoma* ) OR TITLE-ABS-KEY ( myoma* ) OR TITLE-ABS-KEY ( fibroid* ) OR TITLE-ABS-KEY ( "fibroid tumor*" ) ) |  |
| **#5** | Intravascular [ALL] | | “intravascular*”[TW] | TITLE-ABS-KEY(intravascular*) |  |
|  | Intravascular [MH] | | - | - |  |
| **#6** | Intravenous [ALL] | | “intraven*”[TW] | TITLE-ABS-KEY(intraven*) |  |
|  | Intravenous [MH] | | - | - |  |
| **#3** | Leiomyoma [ALL] OR myoma [ALL] OR leiomyomatosis [ALL] OR fibroid [ALL] | | ("leiomyoma*"[TW] OR "myoma*"[TW] OR "fibroid*"[TW] OR "fibroid tumor*"[TW] OR ("leiomyoma"[MH] OR "myoma"[MH] OR "leiomyomatosis"[MH])) | TITLE-ABS-KEY(leiomyoma*) OR TITLE-ABS-KEY(myoma*) OR TITLE-ABS-KEY(fibroid*) OR TITLE-ABS-KEY("fibroid tumor*") |  |
| **#8** | #5 OR #6 AND #3 | |  | ( TITLE-ABS-KEY ( intravascular* ) ) OR ( TITLE-ABS-KEY ( intraven* ) ) AND ( TITLE-ABS-KEY ( leiomyoma* ) OR TITLE-ABS-KEY ( myoma* ) OR TITLE-ABS-KEY ( fibroid* ) OR TITLE-ABS-KEY ( "fibroid tumor*" ) ) |  |
| **#9** | Disseminated [ALL] | | “disseminat*”[TW] | TITLE-ABS-KEY(disseminat*) |  |
|  | Disseminated [MH] OR Dissemination [MH] | | - |  |  |
| **#10** | Diffused [ALL] | | “diffuse*”[TW] | TITLE-ABS-KEY(diffuse*) |  |
|  | Diffused [MH] OR Diffusion [MH] | | - |  |  |
| **#11** | Peritoneal[ALL] | | “periton*”[TW] | TITLE-ABS-KEY(periton*) |  |
|  | Peritoneal[MH] | | "peritoneum"[MAJR] |  |  |
| **#3** | Leiomyoma [ALL] OR myoma [ALL] OR leiomyomatosis [ALL] OR fibroid [ALL] | | ("leiomyoma*"[TW] OR "myoma*"[TW] OR "fibroid*"[TW] OR "fibroid tumor*"[TW] OR ("leiomyoma"[MH] OR "myoma"[MH] OR "leiomyomatosis"[MH])) | TITLE-ABS-KEY(leiomyoma*) OR TITLE-ABS-KEY(myoma*) OR TITLE-ABS-KEY(fibroid*) OR TITLE-ABS-KEY("fibroid tumor*") |  |
| **#12** | (#9 OR #10) AND #11 AND #3 | |  | ( ( TITLE-ABS-KEY ( disseminat* ) ) OR ( TITLE-ABS-KEY ( diffuse* ) ) ) AND ( TITLE-ABS-KEY ( periton* ) ) AND ( TITLE-ABS-KEY ( leiomyoma* ) OR TITLE-ABS-KEY ( myoma* ) OR TITLE-ABS-KEY ( fibroid* ) OR TITLE-ABS-KEY ( "fibroid tumor*" ) ) |  |
| **#13** | Parasitic [ALL] | | “parasit*”[TW] | TITLE-ABS-KEY(parasit*) |  |
|  | Parasitic [MH] | | - |  |  |
| **#3** | Leiomyoma [ALL] OR myoma [ALL] OR leiomyomatosis [ALL] OR fibroid [ALL] | | ("leiomyoma*"[TW] OR "myoma*"[TW] OR "fibroid*"[TW] OR "fibroid tumor*"[TW] OR ("leiomyoma"[MH] OR "myoma"[MH] OR "leiomyomatosis"[MH])) | TITLE-ABS-KEY(leiomyoma*) OR TITLE-ABS-KEY(myoma*) OR TITLE-ABS-KEY(fibroid*) OR TITLE-ABS-KEY("fibroid tumor*") |  |
| **#15** | #13 AND #3 | |  | ( TITLE-ABS-KEY ( parasit* ) ) AND ( TITLE-ABS-KEY ( leiomyoma* ) OR TITLE-ABS-KEY ( myoma* ) OR TITLE-ABS-KEY ( fibroid* ) OR TITLE-ABS-KEY ( "fibroid tumor*" ) ) |  |
| **#16** | #4 OR #8 OR #12 OR #15 | |  | ( ( TITLE-ABS-KEY ( benign* ) AND TITLE-ABS-KEY ( metastas* ) ) OR ( TITLE-ABS-KEY ( intravascular* ) OR TITLE-ABS-KEY ( intraven* ) ) OR ( ( TITLE-ABS-KEY ( disseminat* ) OR TITLE-ABS-KEY ( diffuse* ) ) AND TITLE-ABS-KEY ( periton* ) ) OR ( TITLE-ABS-KEY ( parasit* ) ) ) AND ( TITLE-ABS-KEY ( leiomyoma* ) OR TITLE-ABS-KEY ( myoma* ) OR TITLE-ABS-KEY ( fibroid* ) OR TITLE-ABS-KEY ( "fibroid tumor*" ) ) |  |

| **Supplementary Table S3: Web of Science search strategy** | | | | | |
| --- | --- | --- | --- | --- | --- |
| **Search date:** | | **17/01/2024** | |  |  |
| **#Number** | **Term #1** | | **Scopus search line** | **Translated search line to Web of Science** | **# Reports** |
| **#1** | Benign [ALL] | | TITLE-ABS-KEY(benign*) | TS=(benign*) |  |
|  | Benign [MH] | | - |  |  |
| **#2** | Metastasizing [ALL] | | TITLE-ABS-KEY(metastas*) | TS=(metastas*) |  |
|  | Metastasis [MH] | | - |  |  |
| **#3** | Leiomyoma [ALL] OR myoma [ALL] OR leiomyomatosis [ALL] OR fibroid [ALL] | | TITLE-ABS-KEY(leiomyoma*) OR TITLE-ABS-KEY(myoma*) OR TITLE-ABS-KEY(fibroid*) OR TITLE-ABS-KEY("fibroid tumor*") | TS=(leiomyoma* OR myoma* OR fibroid* OR "fibroid tumor*") |  |
|  |  | | - |  |  |
| **#4** | #1 AND #2 AND #3 | | ( TITLE-ABS-KEY ( benign* ) ) AND ( TITLE-ABS-KEY ( metastas* ) ) AND ( TITLE-ABS-KEY ( leiomyoma* ) OR TITLE-ABS-KEY ( myoma* ) OR TITLE-ABS-KEY ( fibroid* ) OR TITLE-ABS-KEY ( "fibroid tumor*" ) ) | ((TS=(benign*)) AND TS=(metastas*)) AND TS=(leiomyoma* OR myoma* OR fibroid* OR "fibroid tumor*") |  |
| **#5** | Intravascular [ALL] | | TITLE-ABS-KEY(intravascular*) | TS=(intravascular*) |  |
|  | Intravascular [MH] | | - |  |  |
| **#6** | Intravenous [ALL] | | TITLE-ABS-KEY(intraven*) | TS=(intraven*) |  |
|  | Intravenous [MH] | | - |  |  |
| **#3** | Leiomyoma [ALL] OR myoma [ALL] OR leiomyomatosis [ALL] OR fibroid [ALL] | | TITLE-ABS-KEY(leiomyoma*) OR TITLE-ABS-KEY(myoma*) OR TITLE-ABS-KEY(fibroid*) OR TITLE-ABS-KEY("fibroid tumor*") | TS=(leiomyoma* OR myoma* OR fibroid* OR "fibroid tumor*") |  |
| **#8** | #5 OR #6 AND #3 | | ( TITLE-ABS-KEY ( intravascular* ) ) OR ( TITLE-ABS-KEY ( intraven* ) ) AND ( TITLE-ABS-KEY ( leiomyoma* ) OR TITLE-ABS-KEY ( myoma* ) OR TITLE-ABS-KEY ( fibroid* ) OR TITLE-ABS-KEY ( "fibroid tumor*" ) ) | ((TS=(intravascular*)) OR TS=(intraven*)) AND TS=(leiomyoma* OR myoma* OR fibroid* OR "fibroid tumor*") |  |
| **#9** | Disseminated [ALL] | | TITLE-ABS-KEY(disseminat*) | TS=(disseminat*) |  |
|  | Disseminated [MH] OR Dissemination [MH] | |  |  |  |
| **#10** | Diffused [ALL] | | TITLE-ABS-KEY(diffuse*) | TS=(diffuse*) |  |
|  | Diffused [MH] OR Diffusion [MH] | |  |  |  |
| **#11** | Peritoneal[ALL] | | TITLE-ABS-KEY(periton*) | TS=(periton*) |  |
|  | Peritoneal[MH] | |  |  |  |
| **#3** | Leiomyoma [ALL] OR myoma [ALL] OR leiomyomatosis [ALL] OR fibroid [ALL] | | TITLE-ABS-KEY(leiomyoma*) OR TITLE-ABS-KEY(myoma*) OR TITLE-ABS-KEY(fibroid*) OR TITLE-ABS-KEY("fibroid tumor*") | TS=(leiomyoma* OR myoma* OR fibroid* OR "fibroid tumor*") |  |
| **#12** | (#9 OR #10) AND #11 AND #3 | | ( ( TITLE-ABS-KEY ( disseminat* ) ) OR ( TITLE-ABS-KEY ( diffuse* ) ) ) AND ( TITLE-ABS-KEY ( periton* ) ) AND ( TITLE-ABS-KEY ( leiomyoma* ) OR TITLE-ABS-KEY ( myoma* ) OR TITLE-ABS-KEY ( fibroid* ) OR TITLE-ABS-KEY ( "fibroid tumor*" ) ) | ((TS=(disseminat*)) OR TS=(diffuse*)) AND TS=(periton*) AND TS=(leiomyoma* OR myoma* OR fibroid* OR "fibroid tumor*") |  |
| **#13** | Parasitic [ALL] | | TITLE-ABS-KEY(parasit*) | TS=(parasit*) |  |
|  | Parasitic [MH] | |  |  |  |
| **#3** | Leiomyoma [ALL] OR myoma [ALL] OR leiomyomatosis [ALL] OR fibroid [ALL] | | TITLE-ABS-KEY(leiomyoma*) OR TITLE-ABS-KEY(myoma*) OR TITLE-ABS-KEY(fibroid*) OR TITLE-ABS-KEY("fibroid tumor*") | TS=(leiomyoma* OR myoma* OR fibroid* OR "fibroid tumor*") |  |
| **#15** | #13 AND #3 | | ( TITLE-ABS-KEY ( parasit* ) ) AND ( TITLE-ABS-KEY ( leiomyoma* ) OR TITLE-ABS-KEY ( myoma* ) OR TITLE-ABS-KEY ( fibroid* ) OR TITLE-ABS-KEY ( "fibroid tumor*" ) ) | (TS=(parasit*)) AND TS=(leiomyoma* OR myoma* OR fibroid* OR "fibroid tumor*") |  |
| **#16** | #4 OR #8 OR #12 OR #15 | | ((TITLE-ABS-KEY(benign*)) AND (TITLE-ABS-KEY(metastas*)) AND (TITLE-ABS-KEY(leiomyoma*) OR TITLE-ABS-KEY(myoma*) OR TITLE-ABS-KEY(fibroid*) OR TITLE-ABS-KEY("fibroid tumor*"))) OR (((TITLE-ABS-KEY(intravascular*)) OR (TITLE-ABS-KEY(intraven*))) AND (TITLE-ABS-KEY(leiomyoma*) OR TITLE-ABS-KEY(myoma*) OR TITLE-ABS-KEY(fibroid*) OR TITLE-ABS-KEY("fibroid tumor*"))) OR (((TITLE-ABS-KEY(disseminat*)) OR (TITLE-ABS-KEY(diffuse*))) AND (TITLE-ABS-KEY(periton*)) AND (TITLE-ABS-KEY(leiomyoma*) OR TITLE-ABS-KEY(myoma*) OR TITLE-ABS-KEY(fibroid*) OR TITLE-ABS-KEY("fibroid tumor*"))) OR ((TITLE-ABS-KEY(parasit*)) AND (TITLE-ABS-KEY(leiomyoma*) OR TITLE-ABS-KEY(myoma*) OR TITLE-ABS-KEY(fibroid*) OR TITLE-ABS-KEY("fibroid tumor*"))) | #4 OR #8 OR #12 OR #15 |  |

| **Supplementary Table S4: Disseminated Peritoneal Leiomyomatosis (DPL) group data** | | | | | | | | | | | |
| --- | --- | --- | --- | --- | --- | --- | --- | --- | --- | --- | --- |
|  | **References** | **Year** | **Type of article** | **Age of diagnosis** | **Symptoms and signs** | **Diagnostic tool** | **Previous surgery** | **Interval time first surgery - diagnosis** | **Surgery for ESLs** | **Hormonal treatment** | **Outcomes** |
| 1 | Gaichies L et al. | 2017 | CS^1^ | 78 | Compression of adjacent organs; Pelvic mass | CT^3^ | Hysterectomy | 252 | Non-gynaecological surgery | No | No symptoms |
| 2 |  |  |  | 50 | Asymptomatic | Surgery | No | - | Non-gynaecological surgery | No | Size mass decreased |
| 3 | Keskin G et al. | 2013 | CR^2^ | 37 | Abdominal/pelvic pain; Pelvic mass | US^4^ | No | - | No | GnRH^6^ analogues | Size mass decreased |
| 4 | Qadir SY et al. | 2020 | CR | 38 | Abdominal/pelvic pain; Pelvic mass | Surgery | No | - | Non-gynaecological surgery | No | No symptoms |
| 5 | Toriyama A et al. | 2013 | CR | 42 | Pelvic mass | CT | No | - | Hysterectomy | No | Unknown |
| 6 | Batton KA et al. | 2018 | CR | 35 | Dyspnea; Abdominal distension | CT | No | - | No | GnRH analogues | Size mass decreased |
| 7 | Awonuga AO et al. | 2008 | CS | 54 | Abdominal/pelvic pain; Pelvic mass | CT | Hysterectomy | 36 | Non-gynaecological surgery | Others | No symptoms |
| 8 | Thiry T et al. | 2013 | CR | 45 | Vaginal bleeding | CT | Myomectomy | 36 | Non-gynaecological surgery | No | No symptoms |
| 9 | Sharma JB et al. | 2017 | CR | 30 | Abdominal distension | CT | Myomectomy | 84 | Hysterectomy | No | No symptoms |
| 10 | Ye Z et al. | 2022 | CR | 43 | Pelvic mass | US | Hysterectomy | 96 | Non-gynaecological surgery | GnRH analogues | No symptoms |
| 11 | Martins Jordão et al. | 2019 | CR | 46 | Pelvic mass | US | No | - | Non-gynaecological surgery | No | No symptoms |
| 12 | Rosica G et al. | 2011 | CR | 42 | Vaginal bleeding | US | No | - | Hysterectomy | No | No symptoms |
| 13 | Talebian Yazdi et al. | 2010 | CR | 50 | Abdominal/pelvic pain | US | No | - | Non-gynaecological surgery | No | Unknown |
| 14 | Lamarca M et al. | 2012 | CR | 37 | Abdominal distension | US | No | - | Hysterectomy | GnRH analogues | Death |
| 15 | Takeda T et al. | 2008 | CR | 68 | Pelvic mass | RM^5^ | No | - | Hysterectomy | Others | Size mass decreased |
| 16 | Anila KR et al. | 2013 | CR | 35 | Compression of adjacent organs; Abdominal distension | US | Hysterectomy | 36 | Non-gynaecological surgery | No | Unknown |
| 17 | Liu C et al. | 2020 | CR | 48 | Abdominal distension | RM | Myomectomy | 72 | Partial hysterectomy | No | No symptoms |
| 18 | Aruh L et al. | 1993 | CR | 39 | Abdominal/pelvic pain; Pelvic mass | - | Hysterectomy | - | No | GnRH analogues | No symptoms & Size mass decreased |
| 19 | Rieker RJ et al. | 2013 | CS | 50 | Asymptomatic | Surgery | Partial hysterectomy | 7 | Non-gynaecological surgery | No | Unknown |
| 20 |  | 2013 |  | 55 | Abdominal distension | CT | Myomectomy | 252 | Hysterectomy | Others | Unknown |
| 21 | Altinok G et al. | 2000 | CR | 50 | Abdominal distension | US | No | - | Hysterectomy | No | No symptoms |
| 22 | Jindal R et al. | 2022 | CR | 58 | Abdominal/pelvic pain | - | Hysterectomy | - | No | Others | No symptoms |
| 23 | Sharma P et al. | 2004 | CR | 55 | Abdominal distension | US | Hysterectomy | 120 | Non-gynaecological surgery | No | Unknown |
| 24 | Mueller F et al. | 2012 | CR | 41 | Asymptomatic | CT | Myomectomy | 36 | Myomectomy | No | No symptoms |
| 25 | Miyake T et al. | 2008 | CR | 36 | Asymptomatic | RM | Myomectomy | 72 | Myomectomy | GnRHa | No symptoms |
| 26 | Kouakou F et al. | 2013 | CR | 35 | Asymptomatic | Surgery | Cesarean section | - | Non-gynaecological surgery | No | No symptoms |
| 27 | Della Corte et al. | 2018 | CR | 53 | Vaginal bleeding | RM | Myomectomy | 120 | Hysterectomy | No | Unknown |
| 28 | Yuri T et al. | 2015 | CR | 30 | Asymptomatic | CT | Non-gynaecological surgery | 72 | Myomectomy | No | No symptoms |
| 29 | Arneja SK et al. | 2011 | CR | 18 | Abdominal/pelvic pain | US | Myomectomy | 36 | Hysterectomy | No | No symptoms |
| 30 | Nappi C et al. | 2006 | CR | 27 | Abdominal/pelvic pain | US | No | - | Myomectomy | No | Unknown |
| 31 | Bogusiewicz M et al. | 2013 | CR | 42 | Abdominal/pelvic pain | US | Hysterectomy | 26 | Myomectomy | No | Unknown |
| 32 | Tan CH et al. | 2012 | CR | 44 | Abdominal/pelvic pain; Pelvic mass | CT | Myomectomy | 24 | Myomectomy | No | No symptoms |
| 33 | Liu JY et al. | 2009 | CR | 36 | Abdominal/pelvic pain; Pelvic mass | US | Myomectomy | 48 | Non-gynaecological surgery | No | Unknown |
| 34 | DeAngelis SL et al. | 2009 | CR | 40 | Asymptomatic | - | Cesarean section | 30 | Hysterectomy | No | Unknown |
| 35 | Raspagliesi F et al. | 1996 | CS | 26 | Abdominal/pelvic pain | US | Myomectomy | 4 | Non-gynaecological surgery | No | Less symptoms |
| 36 |  | 1996 |  | 48 | Asymptomatic | US | Hysterectomy | 4 | Non-gynaecological surgery | No | Death |
| 37 | Strinić T et al. | 2000 | CR | 66 | Abdominal/pelvic pain; Pelvic mass | US | No | - | Hysterectomy | No | No symptoms |
| 38 | Komatsu M et al. | 1996 | CR | 58 | Abdominal/pelvic pain | Surgery | Hysterectomy | 120 | Non-gynaecological surgery | No | No symptoms |
| 39 | Nigojevic S et al. | 1997 | CR |  | Abdominal/pelvic pain | US | Hysterectomy | - | Myomectomy | No | Unknown |
| 40 | Barone A et al. | 2014 | CR | 32 | Abdominal/pelvic pain; Pelvic mass | US | Myomectomy | 24 | Non-gynaecological surgery | No | Unknown |
| 41 | Papadatos D et al. | 1996 | CR | 48 | Abdominal/pelvic pain; Abdominal distension | US | Hysterectomy | 156 | Non-gynaecological surgery | Others | Size mass decreased |
| 42 | Ramesh L et al. | 2005 | CR | 34 | Vaginal bleeding | Surgery | Non-gynaecological surgery | 84 | Myomectomy | No | No symptoms |
| 43 | Bourgain C et al. | 1994 | CR | 43 | Abdominal/pelvic pain; Abdominal distension | CT | No | - | Hysterectomy | No | Size mass decreased |
| 44 | Karuppaswamy J et al. | 2003 | CR | 45 | Abdominal/pelvic pain; Vaginal bleeding | Surgery | No | - | Hysterectomy | No | Unknown |
| 45 | Ando H et al. | 2017 | CR | 40 | Abdominal/pelvic pain | CT | Myomectomy | 36 | Hysterectomy | No | Size mass decreased |
| 46 | Morizaki A et al. | 1999 | CR | 33 | Abdominal/pelvic pain | Surgery | Myomectomy | 12 | Myomectomy | No | Death |
| 47 | Kökçü A et al. | 1994 | CR | 51 | Compression of adjacent organs; Pelvic mass | Surgery | No | - | No | No | No symptoms |
| 48 | Qin T et al. | 2023 | CR | 46 | Abdominal/pelvic pain | CT | Hysterectomy | 24 | Non-gynaecological surgery | No | No symptoms |
| 49 | Ström H et al. | 1981 | CR | 43 | Vaginal bleeding | Surgery | Non-gynaecological surgery | 12 | Hysterectomy | Others | No symptoms |
| 50 | Hales HA et al. | 1992 | CR | 29 | Abdominal/pelvic pain; Pelvic mass | Surgery | Myomectomy | 12 | Hysterectomy | Others | Size mass decreased |
| 51 | Crosland DB et al. | 1973 | CR | 29 | Asymptomatic | Surgery | No | - | Non-gynaecological surgery | No | Size mass decreased |
| 52 | McCarthy CM et al. | 2017 | CR | 43 | Abdominal/pelvic pain | Surgery | Hysterectomy | 18 | Non-gynaecological surgery | No | Unknown |
| 53 | Braun W et al. | 1994 | CR | 54 | Bone-related | CT | Hysterectomy | - | Non-gynaecological surgery | No | No symptoms |
| 54 | Fujii S et al. | 1980 | CR | 30 | Pelvic mass | Surgery | No | - | Non-gynaecological surgery | No | Unknown |
| 55 | Emoto I et al. | 2018 | CR | 49 | Abdominal/pelvic pain | Surgery | Hysterectomy | 6 | Non-gynaecological surgery | GnRHa | Death |
| 56 | Nogales FF J et al. | 1978 | CR | 34 | Asymptomatic | Surgery | Cesarean section | 0 | Hysterectomy | No | Unknown |
| 57 | Buhusayyen HE et al. | 2022 | CR | 62 | Abdominal/pelvic pain; Abdominal distension | CT | Hysterectomy | 204 | Non-gynaecological surgery | Others | Unknown |
| 58 | Chiu HC et al. | 2018 | CR | 61 | Abdominal/pelvic pain; Pelvic mass | CT | Hysterectomy | 252 | Non-gynaecological surgery | Others | Death |
| 59 | Morgan ED et al. | 2022 | CR | 23 | Asymptomatic | Surgery | Cesarean section | - | Cesarean section | No | No symptoms & Size mass decreased |
| 60 | Bayrak S et al. | 2014 | CR | 49 | Abdominal/pelvic pain; Abdominal distension | Surgery | No | - | Non-gynaecological surgery | No | Unknown |
| 61 | Navarro AS et al. | 2022 | CR | 34 | Abdominal/pelvic pain | CT | Myomectomy | 84 | Non-gynaecological surgery | Ulipristal acetate | No symptoms & Size mass decreased |
| 62 | Huang SF et al. | 2021 | CR | 49 | Abdominal/pelvic pain; Pelvic mass | US | Myomectomy | 156 | Non-gynaecological surgery | No | Size mass decreased & Less symptoms |
| 63 | Yang JW et al. | 2021 | CR | 33 | Abdominal/pelvic pain; Abdominal distension | CT | Hysterectomy | 36 | No | Others | No symptoms & Size mass decreased |
| 64 | La Greca et al. | 2021 | CR | 49 | Abdominal/pelvic pain | US | Myomectomy | 96 | Non-gynaecological surgery | No | No symptoms & Size mass decreased |
| 65 | Aydın A et al. | 2019 | CR | 36 | Abdominal/pelvic pain | CT | No | - | Non-gynaecological surgery | No | No symptoms & Size mass decreased |
| 66 | Ferrario L et al. | 2018 | CR | 36 | Abdominal/pelvic pain; Abdominal distension | RM | Myomectomy | 132 | Non-gynaecological surgery | No | No symptoms |
| 67 | Xu S et al. | 2019 | CR | 47 | Pelvic mass | US | Myomectomy | 120 | Non-gynaecological surgery | Others | No symptoms & Size mass decreased |
| 68 | Julien C et al. | 2018 | CR | 53 | Abdominal/pelvic pain; Abdominal distension | CT | No | - | Non-gynaecological surgery | Others | No symptoms & Size mass decreased |
| 69 | Syed M et al. | 2018 | CR | 40 | Abdominal/pelvic pain | US | Hysterectomy | 36 | Non-gynaecological surgery | No | Unknown |
| 70 | Momtahan M et al. | 2013 | CR | 29 | Abdominal/pelvic pain; Pelvic mass | - | Hysterectomy | 72 | Myomectomy | No | Unknown |
| 71 | Jeyarajah S et al. | 2011 | CR | 69 | Compression of adjacent organs; Abdominal distension | CT | Myomectomy | 240 | Myomectomy | No | No symptoms |
| 72 | Qin T et al. | 2024 | CR | 31 | Abdominal/pelvic pain; Abdominal distension | US | Myomectomy | 24 | Myomectomy | No | No symptoms |
| 73 | Khangar B et al. | 2017 | CR | 42 | Abdominal/pelvic pain; Pelvic mass | US | Hysterectomy | 72 | Non-gynaecological surgery | No | Unknown |
| 74 | Izi et al. | 2023 | CR | 41 | Pelvic mass | US | Myomectomy | 60 | Non-gynaecological surgery | No | No symptoms |
| 75 | Wen C et al. | 2022 | CR | 72 | Abdominal/pelvic pain; Pelvic mass | CT | Hysterectomy | 24 | Non-gynaecological surgery | No | No symptoms |
| 76 | Huang S. et al. | 2021 | CR | 49 | Abdominal/pelvic pain | CT | Hysterectomy | 24 | Non-gynaecological surgery | No | Less symptoms |
| 77 | Kinci M et al. | 2021 | CR | 43 | Abdominal/pelvic pain | CT | Myomectomy | 24 | Non-gynaecological surgery | No | Unknown |
| 78 | Gupta et al. | 2019 | CR | 31 | Abdominal/pelvic pain; Abdominal distension | US | Myomectomy | 60 | Non-gynaecological surgery | No | Unknown |
| 79 | Ferrario et al. | 2018 | CR | 36 | Abdominal/pelvic pain; Abdominal distension | RM | Myomectomy | 132 | Myomectomy | No | No symptoms |
| 80 | Gao et al. | 2018 | CR | 25 | Abdominal/pelvic pain | US | No | - | Myomectomy | No | No symptoms |
| 81 | Zhou et al. | 2017 | CR | 64 | Compression of adjacent organs; Pelvic mass | CT | No | - | Hysterectomy | No | No symptoms |
| 82 | Psathas et al. | 2017 | CR | 40 | Abdominal/pelvic pain; Pelvic mass | CT | Myomectomy | 48 | Myomectomy | No | Unknown |
| 83 | Tyan C. et al. | 2015 | CR | 42 | Abdominal/pelvic pain | Surgery | Hysterectomy | 48 | Myomectomy | No | No symptoms |
| 84 | Bayrak et al. | 2014 | CR | 49 | Abdominal/pelvic pain | Surgery | Non-gynaecological surgery | 12 | Non-gynaecological surgery | No | No symptoms |
| 85 | Ng K et al. | 2014 | CR | 43 | Vaginal bleeding | US | Myomectomy | 36 | Myomectomy | No | No symptoms |
| 86 | Michał et al. | 2013 | CR | 42 | Abdominal/pelvic pain | CT | Hysterectomy | 24 | Myomectomy | No | No symptoms |
| 87 | Honemeyer et al. | 2012 | CR | 39 | Asymptomatic | Surgery | No | - | Myomectomy | No | Size mass decreased |
| 88 | Won H et al. | 2011 | CR | 62 | Abdominal/pelvic pain; Pelvic mass | CT | Hysterectomy | 96 | Non-gynaecological surgery | Others | Size mass decreased |
| 89 | Momtahan et al. | 2011 | CR | 29 | Abdominal/pelvic pain | US | Non-gynaecological surgery | 72 | Non-gynaecological surgery | GnRHa | No symptoms |
| 90 | Kim J et al. | 2010 | CR | 68 | Abdominal/pelvic pain | CT | Hysterectomy | 70 | Non-gynaecological surgery | No | Less symptoms |
| 91 | Rasalkar D et al. | 2010 | CR | 31 | Abdominal/pelvic pain | US | Myomectomy | 60 | Non-gynaecological surgery | No | Unknown |
| 92 | Jeyarajah et al. | 2009 | CR | 69 | Abdominal/pelvic pain; Abdominal distension | CT | Myomectomy | 240 | Myomectomy | No | No symptoms |
| 93 | Grimbizis G et al. | 2006 | CR | 40 | Abdominal/pelvic pain; Pelvic mass | US | No | - | Myomectomy | No | No symptoms |
| ^1^CS: case series; ^2^CR: case report; ^3^CT: computed tomography; ^4^US: ultrasound; ^5^MR: magnetic resonance; ^6^GnRHa: gonadotropin releasing hormone analogue. | | | | | | | | | | | |

| **Supplementary Table S5: Parasitic myoma (PM) group data** | | | | | | | | | | | |
| --- | --- | --- | --- | --- | --- | --- | --- | --- | --- | --- | --- |
|  | **References** | **Year** | **Type of article** | **Age of diagnosis** | **Symptoms and signs** | **Diagnostic tool** | **Previous surgery** | **Interval time first surgery - diagnosis** | **Surgery for ESLs** | **Hormonal treatment** | **Outcomes** |
| 1 | Lin J et al. | 2014 | CR | 43 | Thrombosis-related | TC | Hysterectomy | 12 | Non-gynaecological surgery | No | No symptoms |
| 2 | Gaichies L et al. | 2017 | CS | 78 | Compression of adjacent organs; Pelvic mass | TC | Hysterectomy | 252 | Non-gynaecological surgery | No | No symptoms |
| 3 |  | 2017 |  | 50 | Asymptomatic | Surgery | No | - | Non-gynaecological surgery | No | Size mass decreased |
| 4 | Bilyeu SP et al. | 2006 | CR | 51 | Thrombosis-related | TC |  |  | Hysterectomy | No | Less symptoms |
| 5 | Garcés Garcés J et al. | 2023 | CR | 42 | Vaginal bleeding | TC | Myomectomy | 144 | Hysterectomy | GnRHa | No symptoms |
| 6 | Lee HJ et al. | 2008 | CR | 46 | Vaginal bleeding | US |  |  | Hysterectomy | No | No symptoms |
| 7 | Simon AJ et al. | 2014 | CR | 54 | Thrombosis-related | TC | No | - | Hysterectomy | No | No symptoms |
| 8 | Hur JW et al. | 2015 | CR | 42 | Compression of adjacent organs; Pelvic mass | RM | Myomectomy | 168 | Non-gynaecological surgery | No | No symptoms |
| 9 | Harnoy Y et al. | 2015 | CR | 65 | Thrombosis-related | US | Hysterectomy | 180 | Non-gynaecological surgery | No | No symptoms |
| 10 | Tohya T et al. | 2014 | CR | 49 | Pelvic mass | TC | Myomectomy | 168 | Non-gynaecological surgery | No | No symptoms |
| 11 | Arif S et al. | 2006 | CR | 42 | Abdominal/pelvic pain | RM | Hysterectomy | 156 | Non-gynaecological surgery | GnRHa | No symptoms |
| 12 | Keskin G et al. | 2013 | CR | 37 | Abdominal/pelvic pain; Pelvic mass | US | No | - |  | GnRHa | Size mass decreased |
| 13 | Qadir SY et al. | 2020 | CR | 38 | Abdominal/pelvic pain; Pelvic mass | Surgery | No | - | Non-gynaecological surgery | No | No symptoms |
| 14 | Vural C et al. | 2010 | CR | 48 | Dyspnea | TC | No | - | Hysterectomy | No | No symptoms |
| 15 | Esteban JM et al. | 1999 | CR | 72 | Asymptomatic | TC | Hysterectomy | 48 | Non-gynaecological surgery | No | No symptoms |
| 16 | Tirmazy S et al. | 2023 | CR | 41 | Dyspnea | TC | Hysterectomy | 120 | Non-gynaecological surgery | Others | No symptoms |
| 17 | Nam MS et al. | 2003 | CR | 46 | Abdominal/pelvic pain; Pelvic mass | TC | No | - | Hysterectomy | No | No symptoms |
| 18 | Bodner-Adler B et al. | 2009 | CR | 30 | Vaginal bleeding | US | Myomectomy | 12 | Hysterectomy | GnRHa | No symptoms |
| 19 | Tan Y et al. | 2024 | CS | 45 | Vaginal bleeding | US | Cesarean section | 228 | Partial hysterectomy | No | No symptoms |
| 20 |  | 2024 |  | 53 | Compression of adjacent organs; Abdominal distension | US | No | - | Hysterectomy | No | No symptoms |
| 21 | Kwon YI et al. | 2006 | CR | 51 | Asthenia | TC | Hysterectomy | 192 | Non-gynaecological surgery | No | No symptoms |
| 22 | Chiang CS et al. | 2018 | CR | 49 | Thrombosis-related | TC | Hysterectomy | 18 | Non-gynaecological surgery | No | No symptoms |
| 23 | Cobellis L et al. | 2014 | CR | 43 | Dyspnea | TC | Myomectomy | 144 | Hysterectomy | No | No symptoms |
| 24 | Lam PM et al. | 2004 | CS | 41 | Pelvic mass | TC | No | - | Hysterectomy | No | No symptoms |
| 25 |  | 2004 |  | 47 | Compression of adjacent organs; Abdominal distension | TC | No | - | Hysterectomy | No | No symptoms |
| 26 | Koh DM et al. | 2000 | CR | 40 | Abdominal/pelvic pain; Vaginal bleeding | TC | Hysterectomy | 84 | Non-gynaecological surgery | Others | No symptoms |
| 27 | Yano M et al. | 2020 | CR | 52 | Abdominal/pelvic pain; Pelvic mass | TC | No | - | Hysterectomy | GnRHa | No symptoms |
| 28 | Cosan Sarbay et al. | 2016 | CR | 49 | Abdominal/pelvic pain; Vaginal bleeding | - | No | - | Hysterectomy | No | Unknown |
| 29 | Toriyama A et al. | 2013 | CR | 42 | Pelvic mass | TC | No | - | Hysterectomy | No | Unknown |
| 30 | Clay TD et al. | 2013 | CR | 40 | Thrombosis-related; Pelvic mass | US | Myomectomy | 5 | Hysterectomy | No | Less symptoms |
| ^1^CS: case series; ^2^CR: case report; ^3^CT: computed tomography; ^4^US: ultrasound; ^5^MR: magnetic resonance; ^6^GnRHa: gonadotropin releasing hormone analogue. | | | | | | | | | | | |

| **Supplementary Table S6: Benign metastatic leiomyoma (BML) group data** | | | | | | | | | | | |
| --- | --- | --- | --- | --- | --- | --- | --- | --- | --- | --- | --- |
|  | **References** | **Year** | **Type of article** | **Age of diagnosis** | **Symptoms and signs** | **Diagnostic tool** | **Previous surgery** | **Interval time first surgery - diagnosis** | **Surgery for ESLs** | **Hormonal treatment** | **Outcomes** |
| 1 | Lin J et al. | 2014 | CR | 43 | Thrombosis-related | CT | Hysterectomy | 12 | Non-gynaecological surgery | No | No symptoms |
| 2 | Lee HJ et al. | 2008 | CR | 46 | Vaginal bleeding | US | No | - | Hysterectomy | No | No symptoms |
| 3 | Hur JW et al. | 2015 | CR | 42 | Compression of adjacent organs; Pelvic mass | RM | Myomectomy | 168 | Non-gynaecological surgery | No | No symptoms |
| 4 | Tohya T et al. | 2014 | CR | 49 | Pelvic mass | CT | Myomectomy | 168 | Non-gynaecological surgery | No | No symptoms |
| 5 | Arif S et al. | 2006 | CR | 42 | Abdominal/pelvic pain | RM | Hysterectomy | 156 | Non-gynaecological surgery | GnRH analogues | No symptoms |
| 6 | Esteban JM et al. | 1999 | CR | 72 | Asymptomatic | CT | Hysterectomy | 48 | Non-gynaecological surgery | No | No symptoms |
| 7 | Tirmazy S et al. | 2023 | CR | 41 | Dyspnea | CT | Hysterectomy | 120 | Non-gynaecological surgery | Others | No symptoms |
| 8 | Bodner-Adler B et al. | 2009 | CR | 30 | Vaginal bleeding | US | Myomectomy | 12 | Hysterectomy | GnRH analogues | No symptoms |
| 9 | Kwon YI et al. | 2006 | CR | 51 | Asthenia | CT | Hysterectomy | 192 | Non-gynaecological surgery | No | No symptoms |
| 10 | Cobellis L et al. | 2014 | CR | 43 | Dyspnea | CT | Myomectomy | 144 | Hysterectomy | No | No symptoms |
| 11 | Koh DM et al. | 2000 | CR | 40 | Abdominal/pelvic pain; Vaginal bleeding | CT | Hysterectomy | 84 | Non-gynaecological surgery | Others | No symptoms |
| 12 | Laban KG et al. | 2016 | CR | 69 | Asymptomatic | Others | No | - | Non-gynaecological surgery | No | No symptoms |
| 13 |  | 2008 |  | 35 | Abdominal/pelvic pain; Pelvic mass | CT | Hysterectomy | 72 | Non-gynaecological surgery | Others | No symptoms |
| 14 | Miyazaki M et al. | 2013 | CR | 43 | Thrombosis-related | CT | Hysterectomy | 96 | No | No | No symptoms & Size mass decreased |
| 15 | Orejola WC et al. | 2014 | CR | 41 | Asymptomatic | CT | Partial hysterectomy | - | Non-gynaecological surgery | No | No symptoms |
| 16 | Lee WY et al. | 2015 | CR | 31 | Abdominal/pelvic pain | CT | Myomectomy | 12 | Non-gynaecological surgery | No | No symptoms |
| 17 | Benetti-Pinto CL et al. | 2006 | CS | 51 | Dyspnea | CT | Myomectomy | 204 | Hysterectomy | GnRH analogues | Unknown |
| 18 |  | 2006 |  | 47 | Dyspnea | CT | Hysterectomy | 60 |  | No | Death |
| 19 | Abell MR et al. | 1975 | CR | 27 | Asymptomatic | Surgery | Non-gynaecological surgery | 12 | Hysterectomy | No | Less symptoms |
| 20 | Tori M et al. | 2008 | CR | 47 | Pelvic mass | US | Partial hysterectomy | 24 | Non-gynaecological surgery | No | No symptoms |
| 21 | Choe YH et al. | 2017 | CR | 52 | Dyspnea | CT | Hysterectomy | 168 | No | No | No symptoms |
| 22 | Le Guen et al. | 2020 | CR | 26 | Dyspnea | Others | Cesarean section | 36 | No | No | Size mass decreased |
| 23 | Mogi A et al. | 2012 | CR | 35 | Dyspnea | CT | Myomectomy | 84 | Non-gynaecological surgery | GnRH analogues | No symptoms |
| 24 | Wentling GK et al. | 2005 | CR | 37 | Dyspnea | - | Myomectomy | 72 | Hysterectomy | GnRH analogues | Size mass decreased |
| 25 | Horstmann JP et al. | 1977 | CR | 30 | Vaginal bleeding | Others | Non-gynaecological surgery | - | Hysterectomy | No | No symptoms |
| 26 | Schwarz EI et al. | 2009 | CR | 44 | Asthenia | Others | Hysterectomy | - | Non-gynaecological surgery | No | Unknown |
| 27 | Bachman D et al. | 1976 | CR | 42 | Asymptomatic | CT | Hysterectomy | 84 | Non-gynaecological surgery | No | Unknown |
| 28 | Yoshida T et al. | 2021 | CR | 53 | Vaginal bleeding | CT | Myomectomy | 240 | Hysterectomy | No | No symptoms |
| 29 | Uchida T et al. | 1992 | CR | 36 | Dyspnea | Others | Myomectomy | 144 | Hysterectomy | No | No symptoms |
| 30 | Tong T et al. | 2023 | CR | 50 | Abdominal/pelvic pain | RM | Hysterectomy | 12 | Non-gynaecological surgery | No | No symptoms |
| 31 | Consamus EN et al. | 2015 | CR | 55 | Thrombosis-related | RM | Hysterectomy | 204 | Non-gynaecological surgery | No | Unknown |
| 32 | Minoda N et al. | 2023 | CR | 59 | Asymptomatic | US | Myomectomy | 324 |  | No | No symptoms |
| 33 | Fu Y et al. | 2012 | CR | 46 | Asymptomatic | CT | Hysterectomy | 60 | Non-gynaecological surgery | No | No symptoms |
| 34 | Aoki K et al. | 2020 | CR | 48 | Bone-related | RM | Hysterectomy | 168 | No | GnRH analogues | Size mass decreased |
| 35 | Tavecchio L et al. | 1999 | CR | 63 | Asymptomatic | CT | Hysterectomy | 288 | Non-gynaecological surgery | No | Unknown |
| 36 | Canzonieri V et al. | 1990 | CR | 46 | Asymptomatic | Others | Hysterectomy | 84 | Non-gynaecological surgery | No | Size mass decreased |
| 37 | Beck MM et al. | 2012 | CR | 30 | Vaginal bleeding | CT | Hysterectomy | 60 | Non-gynaecological surgery | Others | Size mass decreased |
| 38 | Yoon J et al. | 2017 | CR | 48 | Dyspnea | Others | Myomectomy | 96 | Hysterectomy | No | Less symptoms |
| 39 | Funakoshi Y et al. | 2004 | CR | 77 | Asymptomatic | Others | Hysterectomy | 144 | Non-gynaecological surgery | No | Size mass decreased |
| 40 | Taveira-DaSilva AM et al. | 2012 | CR | 32 | Asymptomatic | CT | No | - | Myomectomy | Ulipristal acetate | Size mass decreased & Less symptoms |
| 41 | Lipton JH et al. | 1987 | CR | 30 | Dyspnea; Pelvic mass | Others | No | - | Hysterectomy | Others | Less symptoms |
| 42 | Sabatini R et al. | 2002 | CR | 45 | Dyspnea; Vaginal bleeding | Others | No | - | Hysterectomy | GnRH analogues | Size mass decreased & Less symptoms |
| 43 | Silva I et al. | 2012 | CR | 50 | Asymptomatic | Others | Hysterectomy | 192 | Non-gynaecological surgery | GnRH analogues | No symptoms |
| 44 | Challa R et al. | 2010 | CR | 48 | Dyspnea | Others | Hysterectomy | 132 | Non-gynaecological surgery | Others | Size mass decreased & Less symptoms |
| 45 | Rao AV et al. | 2008 | CR | 49 | Compression of adjacent organs | Others | Hysterectomy | 180 | No | No | No symptoms |
| 46 | Jeon HW et al. | 2013 | CS | 53 | Pelvic mass | CT | No | - | Hysterectomy | No | No symptoms |
| 47 |  | 2013 |  | 57 | Asymptomatic | CT | Hysterectomy | - | Non-gynaecological surgery | No | No symptoms & Size mass decreased |
| 48 |  | 2013 |  | 56 | Asymptomatic | Others | Hysterectomy | - | Non-gynaecological surgery | No | Size mass decreased |
| 49 | Sapmaz F et al. | 2008 | CR | 41 | Dyspnea | CT | Hysterectomy | 60 |  | No | Less symptoms |
| 50 | Williams M et al. | 2015 | CR | 51 | Abdominal/pelvic pain | US | No | - | Non-gynaecological surgery | GnRH analogues | Unknown |
| 51 | Goto T et al. | 2011 | CR | 44 | Asymptomatic | Others | Myomectomy | 72 | Non-gynaecological surgery | No | Size mass decreased & Less symptoms |
| 52 | Huang L et al. | 2019 | CR | 36 | Asymptomatic | CT | Hysterectomy | - | Non-gynaecological surgery | No | Size mass decreased |
| 53 | Tietze L et al. | 2000 | CR | 46 | Asymptomatic | - | Hysterectomy | 48 | No | GnRH analogues | Size mass decreased |
| 54 | Jiang J et al. | 2018 | CR | 48 | Asymptomatic | CT | Hysterectomy | 96 | No | Others | Death |
| 55 | Takemura G et al. | 1996 | CR | 44 | Dyspnea | Others | Partial hysterectomy | 48 | Non-gynaecological surgery | No | No symptoms & Size mass decreased |
| 56 | Fukunaga M et al. | 2003 | CR | 62 | Asymptomatic | CT | No | - | Hysterectomy | No | Unknown |
| 57 | Chouchane A et al. | 2024 | CR | 43 | Abdominal/pelvic pain; Abdominal distension | US | Myomectomy | 144 | Hysterectomy | No | Unknown |
| 58 | Scutiero G et al. | 2010 | CR | 42 | Vaginal bleeding | CT | Myomectomy |  | Hysterectomy | GnRH analogues | Unknown |
| 59 | Jo JH et al. | 2006 | CR | 39 | Asymptomatic | Others | Myomectomy | 48 | Hysterectomy | Others | Size mass decreased |
| 60 | Berti AF et al. | 2015 | CR | 74 | Bone-related | RM | Hysterectomy | 372 | Non-gynaecological surgery | No | No symptoms & Size mass decreased |
| 61 | Poujade O et al. | 2010 | CR | 55 | Abdominal/pelvic pain; Pelvic mass | CT | Partial hysterectomy | 132 | Non-gynaecological surgery | No | No symptoms & Size mass decreased |
| 62 | Efared B et al. | 2017 | CR | 57 | Dyspnea | CT | Hysterectomy | 108 | - | Others | No symptoms |
| 63 | Karnib M et al. | 2021 | CR | 45 | Asymptomatic | CT | Partial hysterectomy | 24 | Non-gynaecological surgery | Others | Less symptoms |
| 64 | Wongsripuemtet J et al. | 2011 | CR | 32 | Abdominal/pelvic pain; Pelvic mass | Others | Hysterectomy | 36 | Non-gynaecological surgery | Others | Less symptoms |
| 65 | Elmaci İ et al. | 2020 | CR | 34 | Bone-related | RM | No | - | Non-gynaecological surgery | Others | No symptoms & Size mass decreased |
| 66 | Xiao H et al. | 2012 | CR | 46 | Pelvic mass | CT | Hysterectomy | 96 | Non-gynaecological surgery | Others | No symptoms & Size mass decreased |
| 67 | Gan MF et al. | 2013 | CR | 46 | Asthenia | CT | Hysterectomy | 96 | - | No | Unknown |
| 68 | Kang MW et al. | 2011 | CR | 30 | Bone-related | Others | Myomectomy | 36 | Non-gynaecological surgery | No | No symptoms & Size mass decreased |
| 69 | Maskey-Warzęchowska M et al. | 2017 | CR | 56 | Asymptomatic | Others | Hysterectomy | 180 | Non-gynaecological surgery | No | No symptoms & Size mass decreased |
| 70 | Abalo MR et al. | 2020 | CR | 51 | Abdominal/pelvic pain; Abdominal distension | US | No | - | Non-gynaecological surgery | No | No symptoms & Size mass decreased |
| 71 | Alimi F et al. | 2016 | CR | 60 | Asymptomatic | Others | Myomectomy | - | Non-gynaecological surgery | No | Unknown |
| 72 | di Scioscio et al. | 2009 | CR | 64 | Dyspnea | Others | Hysterectomy | 312 | Non-gynaecological surgery | No | Less symptoms |
| 73 | Egberts JH et al. | 2006 | CR | 42 | Abdominal distension | CT | Hysterectomy | 120 | Non-gynaecological surgery | GnRH analogues | No symptoms & Size mass decreased |
| 74 | Adair LB 2n et al. | 2020 | CR | 46 | Asymptomatic | Others | No | - | Hysterectomy | No | Unknown |
| 75 | Chung YH et al. | 2015 | CR | 39 | Abdominal/pelvic pain; Abdominal distension | CT | Hysterectomy | 48 | Non-gynaecological surgery | GnRH analogues | No symptoms & Size mass decreased |
| 76 | Ma H et al. | 2015 | CR | 45 | Asymptomatic | - | Myomectomy | 132 | Non-gynaecological surgery | No | No symptoms & Size mass decreased |
| 77 | Bakkensen JB et al. | 2018 | CR | 46 | Pelvic mass | US | Hysterectomy | 84 | Non-gynaecological surgery | GnRH analogues | Size mass decreased |
| 78 | Whang SG et al. | 2021 | CR | 45 | Vaginal bleeding | CT | Cesarean section | - | Hysterectomy | Others | No symptoms & Size mass decreased |
| 79 | Londero AP et al. | 2008 | CR | 52 | Asymptomatic | Others | Hysterectomy | 48 | Non-gynaecological surgery | Others | No symptoms & Size mass decreased |
| 80 | Ferrie R et al. | 2004 | CR | 31 | Dyspnea | Others | Cesarean section | 5 | Hysterectomy | GnRH analogues | No symptoms |
| 81 | Jo HC et al. | 2018 | CR | 54 | Abdominal distension | Others | No | - | Non-gynaecological surgery | No | No symptoms & Size mass decreased |
| 82 | Wiencek-Weiss AJ et al. | 2016 | CR | 44 | Asymptomatic | Others | Myomectomy | 156 | Non-gynaecological surgery | GnRH analogues | Size mass decreased |
| 83 | Martínez de Mandojana et al. | 2024 | CR | 41 | Bone-related | RM | Myomectomy | 60 | Hysterectomy | GnRH analogues | No symptoms & Size mass decreased |
| 84 | Li Y et al. | 2022 | CR | 37 | Pelvic mass | US | Myomectomy | 120 | Non-gynaecological surgery | GnRH analogues | No symptoms & Size mass decreased |
| 85 | Li Q et al. | 2022 | CR | 48 | Asymptomatic | Others | Myomectomy | 132 | Non-gynaecological surgery | No | No symptoms & Size mass decreased |
| 86 | Chen A et al. | 2018 | CR | 38 | Asymptomatic | CT | Myomectomy | 60 | Non-gynaecological surgery | Others | Size mass decreased |
| 87 | Meddeb M et al. | 2018 | CR | 36 | Asymptomatic | Others | Hysterectomy | 144 | Non-gynaecological surgery | Others | Size mass decreased |
| 88 | Jolissaint JS et al. | 2016 | CR | 46 | Dyspnea | CT | Hysterectomy | 12 | Non-gynaecological surgery | Others | No symptoms & Size mass decreased |
| 89 | Yu R et al. | 2015 | CR | 51 | Asymptomatic | Others | Partial hysterectomy | 0,5 | Non-gynaecological surgery | No | No symptoms & Size mass decreased |
| 90 | Chen S et al. | 2014 | CR | 32 | Dyspnea | CT | Myomectomy | 1 | Non-gynaecological surgery | Others | No symptoms & Size mass decreased |
| 91 | Ponea AM et al. | 2013 | CR | 64 | Dyspnea | Others | Hysterectomy | 24 | Non-gynaecological surgery | No | Size mass decreased & Less symptoms |
| 92 | Li J et al. | 2022 | CR | 54 | Asymptomatic | US | Hysterectomy | 120 | Non-gynaecological surgery | No | No symptoms & Size mass decreased |
| 93 | Chhabra S et al. | 2022 | CR | 38 | Abdominal/pelvic pain | CT | Hysterectomy | 36 | No | Ulipristal acetate | Less symptoms |
| 94 | Ferreira A et al. | 2022 | CR | 42 | Asymptomatic | Others | Hysterectomy | 36 | Non-gynaecological surgery | GnRH analogues | Size mass decreased & Less symptoms |
| 95 | Padhi P et al. | 2021 | CR | 47 | Asymptomatic | CT | Hysterectomy | 120 |  | Others | Size mass decreased |
| 96 | SunneCTioglu A et al. | 2019 | CR | 42 | Abdominal/pelvic pain | Others | Hysterectomy | 108 | Non-gynaecological surgery | GnRH analogues | Size mass decreased |
| 97 | Khan M et al. | 2018 | CR | 47 | Abdominal/pelvic pain | CT | Partial hysterectomy | 36 | Non-gynaecological surgery | Others | No symptoms & Size mass decreased |
| 98 | Pastré J et al. | 2017 | CR | 76 | Dyspnea | Others | Hysterectomy | 48 | Non-gynaecological surgery | Others | No symptoms & Size mass decreased |
| 99 | Aka N et al. | 2016 | CR | 41 | Dyspnea | Others | Myomectomy | 120 | Non-gynaecological surgery | Others | No symptoms & Size mass decreased |
| 100 | Ağaçkiran Y et al. | 2014 | CR | 44 | Abdominal/pelvic pain | CT | Hysterectomy | 168 | Non-gynaecological surgery | No | Unknown |
| 101 | Lim SY et al. | 2012 | CR | 48 | Pelvic mass | US | Hysterectomy | 96 | Non-gynaecological surgery | No | No symptoms & Size mass decreased |
| 102 | Yoon G et al. | 2011 | CR | 34 | Asymptomatic | CT | Myomectomy | 12 | Hysterectomy | Others | Size mass decreased & Less symptoms |
| 103 | Seong G et al. | 2023 | CR | 42 | Bone-related | CT | Hysterectomy | - | Non-gynaecological surgery | GnRH analogues | Size mass decreased & Less symptoms |
| 104 | Brincat MR et al. | 2023 | CR | 47 | Vaginal bleeding | US | No | - | Hysterectomy | Others | No symptoms |
| 105 | Barber E et al. | 2019 | CR | 49 | Asthenia | RM | Hysterectomy | 156 | Non-gynaecological surgery | Others | No symptoms & Size mass decreased |
| 106 | Lee SR et al. | 2017 | CR | 52 | Asymptomatic | Others | Hysterectomy | 120 | No | GnRH analogues | No symptoms & Size mass decreased |
| 107 | Raś R et al. | 2016 | CR | 53 | Abdominal/pelvic pain | CT | Myomectomy | 312 | Partial hysterectomy | No | Unknown |
| 108 | Nakajo M et al. | 2013 | CR | 50 | Asymptomatic | CT | Hysterectomy | 144 | Non-gynaecological surgery | No | Unknown |
| 109 | AlQudah MA et al. | 2022 | CS | 51 | Dyspnea | CT | Myomectomy | 204 | Non-gynaecological surgery | GnRH analogues | Unknown |
| 110 |  | 2022 |  | 47 | Dyspnea | CT | Hysterectomy | 60 | Non-gynaecological surgery | No | Death |
| 111 | Yanagihara T et al. | 2022 | CR | 49 | Dyspnea | CT | Hysterectomy | 108 | Non-gynaecological surgery | Others | No symptoms |
| 112 | Gad MM et al. | 2021 | CR | 46 | Thrombosis-related | CT | Partial hysterectomy | 24 | Non-gynaecological surgery | GnRH analogues | Unknown |
| 113 | Matos F et al. | 2019 | CR | 40 | Abdominal/pelvic pain | RM | No | - | Hysterectomy | GnRH analogues | No symptoms |
| 114 | Mauduit M et al. | 2020 | CR | 70 | Asymptomatic | CT | Hysterectomy | 276 | Non-gynaecological surgery | No | No symptoms |
| 115 | Kołaczyk K et al. | 2015 | CR | 45 | Asymptomatic | Others | Hysterectomy | 60 | Non-gynaecological surgery | GnRH analogues | Unknown |
| 116 | Jain M et al. | 2014 | CR | 40 | Abdominal/pelvic pain; Abdominal distension | CT | Hysterectomy | 60 | Non-gynaecological surgery | No | Size mass decreased |
| 117 | Naito M et al. | 2011 | CR | 50 | Dyspnea | CT | Hysterectomy | 156 | Non-gynaecological surgery | No | No symptoms |
| 118 | Kachhwaha et al. | 2023 | CR | 37 | Bone-related | RM | Hysterectomy | 252 | Non-gynaecological surgery | Others | No symptoms |
| 119 | Li et al. | 2022 | CR | 37 | Pelvic mass | US | Myomectomy | 120 | Non-gynaecological surgery | GnRH analogues | Unknown |
| 120 | Li et al. | 2022 | CR | 54 | Asymptomatic | US | Hysterectomy | 120 | Non-gynaecological surgery | No | Unknown |
| 121 | Alqudah M et al. | 2022 | CS | 51 | Dyspnea | CT | Myomectomy | 204 | Hysterectomy | GnRH analogues | Unknown |
| 122 |  | 2022 |  | 47 | Dyspnea | CT | Hysterectomy | 60 | Non-gynaecological surgery | No | Death |
| 123 | Gosavi et al. | 2022 | CR | 47 | Abdominal/pelvic pain; Abdominal distension | CT | Hysterectomy | - | No | GnRH analogues | Unknown |
| 124 | Whang S et al. | 2021 | CR | 45 | Vaginal bleeding | CT | Cesarean section | - | Non-gynaecological surgery | No | No symptoms |
| 125 | Padhi et al. | 2021 | CR | 37 | Asymptomatic | CT | Hysterectomy | 120 | Non-gynaecological surgery | No | Size mass decreased |
| 126 | Reis Soares et al. | 2020 | CR | 42 | Dyspnea | RM | Hysterectomy | 24 | Non-gynaecological surgery | No | Unknown |
| 127 | Li et al. | 2019 | CR | 48 | Asymptomatic | Others | Myomectomy | 132 | Non-gynaecological surgery | No | No symptoms |
| 128 | Jo H et al. | 2018 | CR | 54 | Compression of adjacent organs; Pelvic mass | CT | No | - | Hysterectomy | No | No symptoms & Size mass decreased |
| 129 | Khan et al. | 2018 | CR | 47 | Dyspnea | CT | Partial hysterectomy | 36 | Non-gynaecological surgery | GnRH analogues | Size mass decreased |
| 130 | Kyriakopoulos et al. | 2018 | CR | 55 | Abdominal/pelvic pain; Pelvic mass | US | Hysterectomy | 156 | Myomectomy | No | No symptoms |
| 131 | Solazzo et al. | 2017 | CR | 57 | Asymptomatic | CT | Myomectomy | 120 | Non-gynaecological surgery | No | No symptoms |
| 132 | Pastré et al. | 2017 | CR | 76 | Dyspnea | CT | Hysterectomy | 48 | Non-gynaecological surgery | Others | No symptoms |
| 133 | Song K et al. | 2017 | CR | 61 | Dyspnea | CT | Hysterectomy | 108 | Non-gynaecological surgery | No | No symptoms |
| 134 | Lee S et al. | 2017 | CR | 52 | Asymptomatic | Others | Hysterectomy | 120 | Non-gynaecological surgery | GnRH analogues | No symptoms & Size mass decreased |
| 135 | Aka et al. | 2016 | CR | 41 | Dyspnea | CT | Myomectomy | 120 | Non-gynaecological surgery | No | No symptoms & Size mass decreased |
| 136 | Elamaran et al. | 2016 | CR | 51 | Dyspnea | CT | Hysterectomy | 132 | Non-gynaecological surgery | No | No symptoms & Size mass decreased |
| 137 | Ouyang et al. | 2015 | CR | 47 | Dyspnea | CT | Myomectomy | 72 | Hysterectomy | No | Unknown |
| 138 | Raś et al. | 2016 | CR | 53 | Abdominal/pelvic pain; Pelvic mass | CT | Myomectomy | 312 | Hysterectomy | No | No symptoms |
| 139 | Kołaczyk et al. | 2015 | CR | 45 | Asymptomatic | Others | Hysterectomy | 84 | Non-gynaecological surgery | No | No symptoms |
| 140 | Chen et al. | 2014 | CR | 32 | Dyspnea | CT | Myomectomy | 10 | Non-gynaecological surgery | No | No symptoms |
| 141 | Hanada et al. | 2013 | CR | 71 | Compression of adjacent organs | CT | Myomectomy | 48 | Non-gynaecological surgery | No | Less symptoms |
| 142 | Zhu et al. | 2012 | CR | 38 | Asymptomatic | CT | Hysterectomy | 96 | Non-gynaecological surgery | No | Unknown |
| 143 | Lim S et al. | 2011 | CR | 48 | Pelvic mass | RM | Hysterectomy | 96 | Non-gynaecological surgery | No | No symptoms |
| 144 | Yoon et al. | 2011 | CR | 34 | Pelvic mass | CT | Myomectomy | 12 | Non-gynaecological surgery | No | No symptoms |
| 145 | Pekçolaklar et al. | 2011 | CR | 50 | Dyspnea | CT | Hysterectomy | 60 | Non-gynaecological surgery | No | Unknown |
| 146 | Fukushima et al. | 2010 | CR | 42 | Dyspnea | CT | Myomectomy | 60 | Non-gynaecological surgery | No | Size mass decreased & Less symptoms |
| 147 | Simon et al. | 2010 | CR | 51 | Compression of adjacent organs | RM | Hysterectomy | 84 | Non-gynaecological surgery | Others | Size mass decreased & Less symptoms |
| 148 | Kapila et al. | 2008 | CR | 42 | Compression of adjacent organs | CT | Myomectomy | 96 | Myomectomy | No | Unknown |
| 149 | Londero A et al. | 2008 | CR | 52 | Abdominal/pelvic pain; Abdominal distension | RM | Hysterectomy | 36 | Non-gynaecological surgery | No | Size mass decreased & Less symptoms |
| 150 | Byung K et al. | 2003 | CR | 41 | Asymptomatic | RM | Hysterectomy | 36 | Non-gynaecological surgery | No | No symptoms |
| 151 | Ferrie et al. | 2001 | CR | 31 | Dyspnea | Others | Cesarean section | 0 | Non-gynaecological surgery | GnRH analogues | No symptoms |
| 152 | Lo, et al. | 2005 | CR | 58 | Pelvic mass | CT | Hysterectomy | 72 | Non-gynaecological surgery | Ulipristal acetate | Size mass decreased |
| 153 | Siddiqui, et al. | 2003 | CR | 76 | Asymptomatic | Others | Hysterectomy | 72 | Non-gynaecological surgery | No | Unknown |
| 154 | AbuRustum, et al. | 1997 | CS | 40 | Vaginal bleeding | CT | Myomectomy | 108 | Non-gynaecological surgery | No | No symptoms & Size mass decreased |
| 155 |  | 1997 |  | 40 | Dyspnea | CT | Hysterectomy | 108 | Non-gynaecological surgery | GnRH analogues | No symptoms & Size mass decreased |
| ^1^CS: case series; ^2^CR: case report; ^3^CT: computed tomography; ^4^US: ultrasound; ^5^MR: magnetic resonance; ^6^GnRHa: gonadotropin releasing hormone analogue. | | | | | | | | | | | |

| **Supplementary Table S7: Intravascular leiomyoma (IVL) group data** | | | | | | | | | | | |
| --- | --- | --- | --- | --- | --- | --- | --- | --- | --- | --- | --- |
|  | **References** | **Year** | **Type of article** | **Age of diagnosis** | **Symptoms and signs** | **Diagnostic tool** | **Previous surgery** | **Interval time first surgery - diagnosis** | **Surgery for ESLs** | **Hormonal treatment** | **Outcomes** |
| 1 | Bilyeu SP et al. | 2006 | CR | 51 | Thrombosis-related | CT | No | - | Hysterectomy | No | Less symptoms |
| 2 | Garcés Garcés J et al. | 2023 | CR | 42 | Vaginal bleeding | CT | Myomectomy | 144 | Hysterectomy | GnRH analogues | No symptoms |
| 3 | Simon AJ et al. | 2014 | CR | 54 | Thrombosis-related | CT | No | - | Hysterectomy | No | No symptoms |
| 4 | Harnoy Y et al. | 2015 | CR | 65 | Thrombosis-related | US | Hysterectomy | 180 | Non-gynaecological surgery | No | No symptoms |
| 5 | Vural C et al. | 2010 | CR | 48 | Dyspnea | CT | No | - | Hysterectomy | No | No symptoms |
| 6 | Nam MS et al. | 2003 | CR | 46 | Abdominal/pelvic pain; Pelvic mass | CT | No | - | Hysterectomy | No | No symptoms |
| 7 | Tan Y et al. | 2024 | CS | 45 | Vaginal bleeding | US | Cesarean section | 228 | Partial hysterectomy | No | No symptoms |
| 8 |  | 2024 |  | 53 | Compression of adjacent organs; Abdominal distension | US | No | - | Hysterectomy | No | No symptoms |
| 9 | Chiang CS et al. | 2018 | CR | 49 | Thrombosis-related | CT | Hysterectomy | 18 | Non-gynaecological surgery | No | No symptoms |
| 10 | Lam PM et al. | 2004 | CS | 41 | Pelvic mass | CT | No | - | Hysterectomy | No | No symptoms |
| 11 |  | 2004 |  | 47 | Compression of adjacent organs; Abdominal distension | CT | No | - | Hysterectomy | No | No symptoms |
| 12 | Yano M et al. | 2020 | CR | 52 | Abdominal/pelvic pain; Pelvic mass | CT | No | - | Hysterectomy | GnRH analogues | No symptoms |
| 13 | Cosan Sarbay et al. | 2016 | CR | 49 | Abdominal/pelvic pain; Vaginal bleeding | - | No | - | Hysterectomy | No | Unknown |
| 14 | Clay TD et al. | 2013 | CR | 40 | Thrombosis-related; Pelvic mass | US | Myomectomy | 5 | Hysterectomy | No | Less symptoms |
| 15 | Negri F et al. | 2022 | CR | 47 | Dyspnea | US | Hysterectomy | - | Non-gynaecological surgery | No | No symptoms |
| 16 | Lo KW et al. | 2001 | CR | 65 | Vaginal bleeding | US | No | - | Hysterectomy | Others | No symptoms |
| 17 | Nishida N et al. | 2004 | CR | 44 | Abdominal/pelvic pain | US | No | - | Hysterectomy | GnRH analogues | No symptoms |
| 18 | Hashiguchi J et al. | 1994 | CR | 48 | Abdominal/pelvic pain | CT | No | - | Hysterectomy | No | Unknown |
| 19 | Marcus SG et al. | 1994 | CR | 45 | Dyspnea | CT | Hysterectomy | 60 | Non-gynaecological surgery | No | No symptoms |
| 20 | Mizoguchi C et al. | 2016 | CR | 50 | Vaginal bleeding | US | No | - | Hysterectomy | Others | No symptoms |
| 21 | Park SY et al. | 2020 | CR | 50 | Abdominal/pelvic pain; Abdominal distension | US | No | - | Hysterectomy | No | No symptoms |
| 22 | Alves AJ et al. | 2016 | CR | 45 | Dyspnea | US | No | - | Hysterectomy | No | No symptoms |
| 23 | Cowie P et al. | 2021 | CR | 77 | Abdominal/pelvic pain; Pelvic mass | US | Cesarean section | 684 | Hysterectomy | No | No symptoms |
| 24 | Sakamoto H et al. | 2004 | CR | 72 | Thrombosis-related | US | Hysterectomy | 360 | Non-gynaecological surgery | No | No symptoms |
| 25 | Wu CK et al. | 2009 | CR | 39 | Dyspnea | US | No | - | Hysterectomy | No | Unknown |
| 26 | Quade BJ et al. | 2002 | CR | 40 | Dyspnea | US | No | - | Hysterectomy | No | Unknown |
| 27 | Okamoto H et al. | 1994 | CR | 48 | Dyspnea | CT | Hysterectomy | 60 | Non-gynaecological surgery | No | Unknown |
| 28 | Saitoh M et al. | 2004 | CR | 47 | Pelvic mass | US | No | - | Hysterectomy | No | No symptoms |
| 29 | Li M et al. | 2019 | CR | 48 | Dyspnea | US | No | - | Hysterectomy | No | No symptoms |
| 30 | Wong YY et al. | 2006 | CR | 54 | Abdominal/pelvic pain | CT | No | - | Hysterectomy | Others | Unknown |
| 31 | Maneyama H et al. | 2016 | CR | 44 | Pelvic mass | CT | No | - | Hysterectomy | No | No symptoms |
| 32 | Han HS et al. | 1998 | CR | 45 | Abdominal/pelvic pain | US | Hysterectomy | 25 | Non-gynaecological surgery | No | Unknown |
| 33 | Bertrand P et al. | 1998 | CR | 41 | Compression of adjacent organs | US | Hysterectomy | 36 | Non-gynaecological surgery | No | No symptoms |
| 34 | Ricci MA et al. | 1995 | CR | 43 | Thrombosis-related; Vaginal bleeding | CT | Myomectomy | 21 | Hysterectomy | No | No symptoms |
| 35 | Bender LC et al. | 2011 | CR | 55 | Dyspnea | RM | Cesarean section | - | Hysterectomy | No | No symptoms |
| 36 | BORLAND DS et al. | 1964 | CR | 62 | Asymptomatic | - | Non-gynaecological surgery | - | Hysterectomy | No | No symptoms |
| 37 | Virzì G et al. | 2007 | CR | 42 | Dyspnea | US | Hysterectomy | 36 | Non-gynaecological surgery | No | No symptoms |
| 38 | Itani Y et al. | 2002 | CR | 47 | Abdominal/pelvic pain; Abdominal distension | Others | Hysterectomy | 36 | Non-gynaecological surgery | No | No symptoms |
| 39 | Rajaii-Khorasani A et al. | 2012 | CR | 25 | Dyspnea | Others | Myomectomy | - | Hysterectomy | GnRH analogues | No symptoms |
| 40 | Arora R et al. | 2010 | CR | 48 | Vaginal bleeding | Surgery | Non-gynaecological surgery | - | Hysterectomy | No | No symptoms |
| 41 | Ahmed M et al. | 2004 | CR | 48 | Compression of adjacent organs | RM | Hysterectomy | 24 | Non-gynaecological surgery | No | Unknown |
| 42 | Ghanem M et al. | 2019 | CR | 48 | Asymptomatic | CT | Hysterectomy | 36 | Non-gynaecological surgery | No | No symptoms |
| 43 | Basso LV et al. | 1984 | CR | 65 | Dyspnea | Others | Hysterectomy | 240 | Non-gynaecological surgery | No | No symptoms |
| 44 | Kawakami S et al. | 1991 | CR | 38 | Vaginal bleeding | CT | Hysterectomy | 16 | Non-gynaecological surgery | No | Unknown |
| 45 | Okamura H et al. | 2011 | CR | 58 | Asymptomatic | US | Hysterectomy | 48 | No | No | No symptoms & Size mass decreased |
| 46 | Rotter AJ et al. | 1991 | CR | 56 | Thrombosis-related | CT | Hysterectomy | 84 | Non-gynaecological surgery | GnRH analogues | Unknown |
| 47 | Timmis AD et al. | 1980 | CR | 46 | Abdominal/pelvic pain | Others | Hysterectomy | 30 | Non-gynaecological surgery | No | Less symptoms |
| 48 | Konrad P et al. | 1989 | CR | 46 | Abdominal/pelvic pain | Others | No | - | Hysterectomy | No | Unknown |
| 49 | Fukuyama A et al. | 2010 | CR | 43 | Pelvic mass | RM | No | - | Hysterectomy | No | No symptoms & Size mass decreased |
| 50 | Pfenniger A et al. | 2021 | CR | 54 | Asymptomatic | US | Hysterectomy | 240 | Non-gynaecological surgery | No | No symptoms & Size mass decreased |
| 51 | Stolf NA et al. | 1999 | CR | 43 | Dyspnea | US | No | - | Non-gynaecological surgery | No | No symptoms & Size mass decreased |
| 52 | Joo HJ et al. | 2012 | CR | 43 | Compression of adjacent organs | RM | Hysterectomy | 72 | Non-gynaecological surgery | No | No symptoms |
| 53 | Brar R et al. | 2018 | CR | 52 | Dyspnea; Pelvic mass | US | No | - | Hysterectomy | No | No symptoms & Size mass decreased |
| 54 | Konishi I et al. | 1987 | CR | 45 | Abdominal/pelvic pain | Surgery | No | - | Hysterectomy | No | No symptoms & Size mass decreased |
| 55 | Siatecka H et al. | 2022 | CR | 50 | Abdominal/pelvic pain | CT | No | - | Hysterectomy | No | Unknown |
| 56 | Schaas CM et al. | 2022 | CR | 44 | Vaginal bleeding | US | Cesarean section | - | Hysterectomy | No | No symptoms & Size mass decreased |
| 57 | Wu X et al. | 2022 | CR | 55 | Asymptomatic | US | No | - | Non-gynaecological surgery | No | Unknown |
| 58 | Reis Soares et al. | 2021 | CR | 42 | Dyspnea | US | Hysterectomy | 24 | Non-gynaecological surgery | No | No symptoms |
| 59 | Magdalena P et al. | 2023 | CR | 47 | Asymptomatic | US | No | - | Non-gynaecological surgery | No | No symptoms & Size mass decreased |
| 60 | Cassol DF et al. | 2023 | CR | 31 | Dyspnea | US | Hysterectomy | 1 | Non-gynaecological surgery | No | No symptoms & Size mass decreased |
| 61 | Konishi H et al. | 2018 | CS | 55 | Pelvic mass | CT | Hysterectomy | 120 |  | Others | Death |
| 62 |  | 2018 |  | 46 | Pelvic mass | CT | No | - | Hysterectomy | No | Size mass decreased & Less symptoms |
| 63 | Ouyang A et al. | 2015 | CR | 42 | Dyspnea | CT | Myomectomy | 72 | Non-gynaecological surgery | No | Unknown |
| 64 | Ceballos B et al. | 2022 | CR | 46 | Abdominal/pelvic pain | CT | Hysterectomy | 48 | Non-gynaecological surgery | No | Unknown |
| 65 | Kılıç Ş et al. | 2022 | CR | 49 | Abdominal/pelvic pain | CT | Myomectomy | 96 | Non-gynaecological surgery | No | No symptoms |
| 66 | Shaked E et al. | 2022 | CR | 47 | Thrombosis-related | US | Partial hysterectomy | 36 | Non-gynaecological surgery | No | No symptoms |
| 67 | Peña A et al. | 2018 | CR | 45 | Thrombosis-related | US | Hysterectomy | 24 | Non-gynaecological surgery | Others | No symptoms |
| 68 | Zeng H et al. | 2016 | CS | 46 | Dyspnea | US | Myomectomy | 156 | Non-gynaecological surgery | No | No symptoms |
| 69 |  | 2016 |  | 45 | Thrombosis-related | US | Myomectomy | 84 | Hysterectomy | No | No symptoms |
| 70 | Abdelghany M et al. | 2014 | CR | 40 | Asymptomatic | US | No | - | Non-gynaecological surgery | No | No symptoms |
| 71 | Li T et al. | 2023 | CR | 46 | Dyspnea | Others | Myomectomy | 60 | Non-gynaecological surgery | No | No symptoms |
| 72 | Cohen D et al. | 2023 | CR | 52 | Dyspnea | CT | No | - | Non-gynaecological surgery | Others | No symptoms |
| 73 | Van Maercke et al. | 2020 | CR | 53 | Dyspnea | CT | No | - | Non-gynaecological surgery | Others | No symptoms |
| 74 | Kikuchi DS et al. | 2023 | CR | 44 | Dyspnea; Abdominal distension | CT | No | - | Non-gynaecological surgery | No | No symptoms |
| 75 | Dolgun ZN et al. | 2015 | CR | 49 | Abdominal/pelvic pain | US | No | - | Hysterectomy | No | Unknown |
| 76 | Chou C. et al. | 2023 | CR | 42 | Abdominal distension | RM |  |  | Hysterectomy | No | No symptoms |
| 77 | Magdalena et al. | 2023 | CR | 47 | Asymptomatic | US | Non-gynaecological surgery | 240 | Non-gynaecological surgery | Others | No symptoms |
| 78 | Iacona G et al. | 2023 | CR | 48 | Asymptomatic | CT | Hysterectomy | 7 | Non-gynaecological surgery | No | No symptoms |
| 79 | Cassol D et al. | 2023 | CR | 31 | Dyspnea | US | Hysterectomy | 0 | Non-gynaecological surgery | No | No symptoms |
| 80 | Vimercati et al. | 2022 | CR | 47 | Abdominal/pelvic pain; Abdominal distension | CT | Myomectomy | 156 | Hysterectomy | No | No symptoms |
| 81 | Lan et al. | 2022 | CR | 31 | Asymptomatic | RM | Myomectomy | 12 | Non-gynaecological surgery | No | No symptoms |
| 82 | Ceballos et al. | 2022 | CR | 46 | Abdominal/pelvic pain | - | Myomectomy | 48 | Non-gynaecological surgery | No | No symptoms |
| 83 | Shaked et al. | 2022 | CR | 47 | Thrombosis-related; Abdominal distension | US | Hysterectomy | 36 | Non-gynaecological surgery | Others | No symptoms |
| 84 | Kim N et al. | 2013 | CR | 43 | Abdominal/pelvic pain; Pelvic mass | CT | Cesarean section | - | Hysterectomy | No | Unknown |
| 85 | Jian et al. | 2010 | CR | 52 | Dyspnea | CT | Myomectomy | 120 | Non-gynaecological surgery | No | Death |
| 86 | Agarwal et al. | 2010 | CR | 50 | Abdominal/pelvic pain | US | No | - | Hysterectomy | GnRH analogues | No symptoms & Size mass decreased |
| 87 | Jiang W. et al. | 2009 | CR | 43 | Dyspnea; Pelvic mass | CT | Partial hysterectomy | 36 | Non-gynaecological surgery | No | No symptoms |
| 88 | Powell J et al. | 2003 | CR | 65 | Abdominal/pelvic pain | RM | Non-gynaecological surgery | 348 | Non-gynaecological surgery | No | No symptoms |
| 89 | Timmis A et al. | 1980 | CR | 46 | Thrombosis-related; Vaginal bleeding | US | Hysterectomy | 36 | Non-gynaecological surgery | No | Size mass decreased & Less symptoms |
| 90 | Iverson L.I et al. | 1983 | CR | 51 | Asthenia | CT | Hysterectomy | 84 | Non-gynaecological surgery | GnRH analogues | No symptoms |
| 91 | Morice et al. | 2001 | CR | 48 | Dyspnea | CT | Hysterectomy | 96 | Non-gynaecological surgery | GnRH analogues | No symptoms |
| 92 | Ferret et al. | 2023 | CR | 45 | Asymptomatic | CT | Hysterectomy | 20 | Non-gynaecological surgery | No | Size mass decreased |
| 93 | Jaiswal, et al. | 2020 | CR | 40 | Abdominal/pelvic pain | CT | Hysterectomy | 96 | Non-gynaecological surgery | No | No symptoms |
| 94 | Longacre, et al. | 1996 | CR | 49 | - | CT | No | 10 | Non-gynaecological surgery | No | No symptoms |
| ^1^CS: case series; ^2^CR: case report; ^3^CT: computed tomography; ^4^US: ultrasound; ^5^MR: magnetic resonance; ^6^GnRHa: gonadotropin releasing hormone analogue. | | | | | | | | | | | |
